# Analysis of causal pathogens of mulberry bacterial blight in samples collected from eight provinces of China using culturomics and metagenomic sequencing methods

**DOI:** 10.3389/fpls.2025.1517050

**Published:** 2025-02-28

**Authors:** Xinpeng Huang, Ting Yuan, Yuxin Huang, Izhar Hyder Qazi, Jiping Liu

**Affiliations:** Guangdong Provincial Key Lab of Agro-Animal Genomics and Molecular Breeding, College of Animal Science, South China Agricultural University, Guangzhou, Guangdong, China

**Keywords:** bacterial blight, climate, culturomics, distribution, metagenomics, mulberry

## Abstract

Mulberry bacterial blight (MBB) is a complex and one of the devastating diseases of mulberry that causes serious reduction in the yield and quality of mulberry. In recent years, the transformation of sericulture industry, mulberry production system, and increasing seedling trade have resulted in the spread of MBB to different parts of China, posing a major economic threat to the farmers and industry. This study investigated the occurrence of MBB in eight provinces of China during years 2023 and 2024. The MBB disease samples were collected and the composition of the MBB pathogenic microbiome was analyzed by combining culturomics and metagenomic sequencing methods. A total of 498 bacterial strains were isolated and identified through culturomics, and then 109 suspected pathogen strains were preliminarily screened based on metagenomic sequencing data. Finally, 10 pathogens including, *Pseudomonas syringae, P. fulva, P. fluorescens, Pantoea ananatis, Pectobacterium parvum, P. carotovorum, Flavobacterium fluviale, Citrobacter portucalensis, Klebsiella grimontii, Stenotrophomonas maltophilia*, were identified through Koch’s postulates. Based on the distribution pattern of pathogens and the changes in the microbiome community of mulberry following infection with *P. syringae*, we infer that *P. syringae*, and *P. fulva* are important pathogens of MBB. In addition, based on the analysis of meteorological data, different bacteria showed adaptability to different environments, leading to differences in the pathogens of MBB under different climate conditions and latitudes. The data presented herein provides a foundation for understanding the occurrence, spatial distribution and pathogenic mechanism of MBB and its major pathogens.

## Graphical Abstract

Cfa: Humid subtropical climate. Cwa: Dry-winter humid subtropical climate. Cwb: Dry-winter subtropical highland climate. SX: Shaanxi province. SC: Sichuan province. YN: Yunnan province. GD: Guangdong province. ZJ: Zhejiang province. JS: Jiangsu province. AH: Anhui province. GX: Guangxi Zhuang autonomous region. TEMP: Monthly average temperature. SSD: Sunshine duration. PRCP: Monthly total precipitation. RH: Relative humidity. WDSP: Wind direction speed. PRES: Station pressure. Arrows indicate positive correlation.

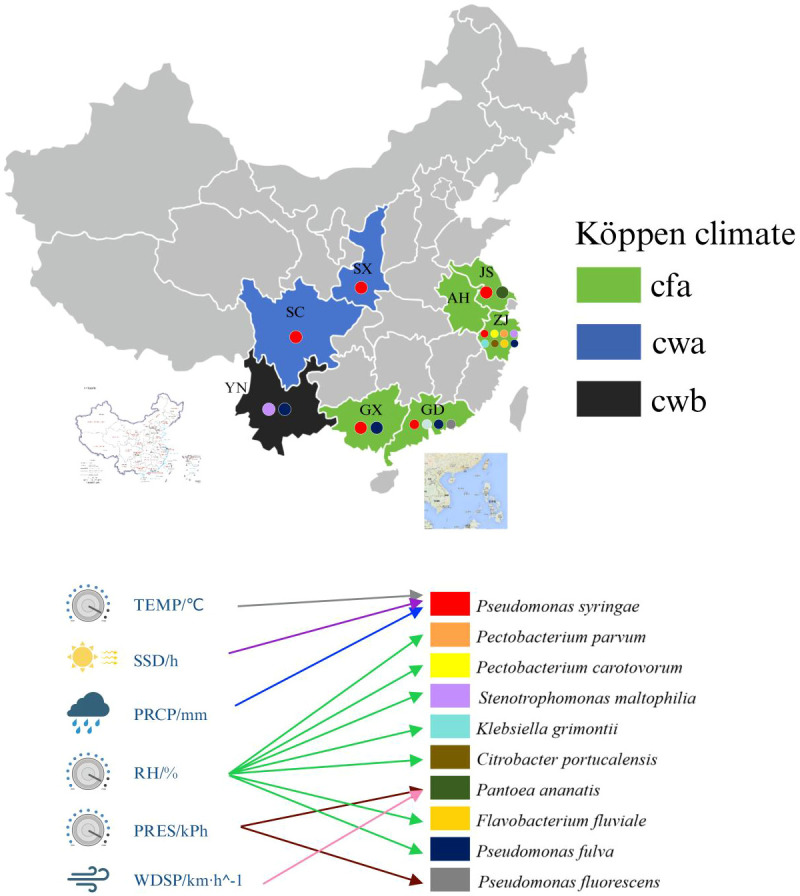

## 1 Introduction

Mulberry is an important economic crop that has been cultivated and used in sericulture in China for over 5000 years (Chen et al., 2022). The stability of the global silk supply chain is highly reliant on healthy growth of mulberry production systems (Memete et al., 2022). In recent years, mulberry bacterial blight (MBB) has emerged as one of the major diseases affecting the growth, yield, and quality of mulberry (Xie et al., 2017). The occurrence of MBB has been reported in several countries including China, India, Iran, Pakistan, Indonesia, and Poland, among others (Krawczyk and Łochyńska, 2020). Although, MBB has been reported in many provinces and different agro-climatic conditions in China, the estimation of its exact economic impact is difficult due to the complexities of cropping practices and sericulture industry itself. However, the estimated direct economic losses are huge in areas where intensive sericulture/moriculture is practiced (Liu Jiping, personal communication).

At present, *Pseudomonas syringae* is generally considered as the main causal pathogen of MBB (Akhtar and Sarwar, 1988; Krawczyk and Łochyńska, 2020). To date, over 50 pathovars of *P. syringae* have been reported worldwide, which are pathogenic to over 180 plant species, including mulberry trees (Krawczyk and Łochyńska, 2020). *P. syringae* can spread through wind and rain. After attachment to the host surface, it quickly causes necrotic spots on the leaves and shoots, causing serious damage to plant health (Donati et al., 2020). Importantly, available evidence shows the diversity of causal pathogens of the MBB, as several other pathogens including *Achromobacter xylosoxidans* (Oksel et al., 2024), *P. oryzihabitans* (Huai et al., 2023), *Xanthomonas phaseoli* (Zárate-Chaves et al., 2024), and *Klebsiella oxytoca* (Huang et al., 2024) have been reported to cause bacterial blight in plants. On the other hand, we now know that bacterial diseases in plants can be caused by co-infection of multiple bacterial species. For instance, recently it was shown that *K. michiganensis* (Luo et al., 2022), *Ralstonia pseudosolanacearum* (Li et al., 2024), *Pantoea ananatis* (Yuan et al., 2023a) can all cause bacterial wilt disease in mulberry trees. In light of this evidence, it is important to investigate the diversity of the causal pathogens of bacterial blight disease of mulberry.

At present, the strategies for prevention and control of MBB are still limited, therefore, research on the diversity of its pathogenic microbiome is very critical for understanding the disease itself. With the rapid development of molecular sequencing technology, metagenomic sequencing and other omics tools are now gaining popularity in the field of plant pathogen diagnosis (Melcher et al., 2014). Compared with the traditional culture-dependent technique, these molecular methods not only provide advantage of simultaneous detection of multiple pathogens, but they are also useful in studying the diversity, structure and composition of the entire plant-associated microbiome in a given sample (Roman-Reyna and Crandall, 2024). These methods also allow exploration and prediction of novel genes and metabolic pathways implicated in pathogenesis of plant diseases (Roman-Reyna and Crandall, 2024). Genomic information can be obtained directly from environmental samples, and many uncultured microbial species can be identified (Xu et al., 2018). Although, genomic analysis-based methods provide rich data, and that the technological advancement has made it possible to extract DNA/RNA from environmental samples with reduced contamination and improved purity, these methods come with some technical limitations. Restrictions in sequencing methods, gene assembly with the risk of chimaera production, and inadequate quality/availability of annotated genes and gene families in the databases are some of the key limitations that lead to genes of unknown functions and consequently to unknown taxa (Sarhan et al., 2019). These problems can easily lead to bias in researchers’ analysis of the microbial samples and incorrect functional prediction of microbial genes. Culturomics, developed by Jean-Christophe Lagier et al. (2015), refers to a high-throughput culture method for obtaining a variety of pure bacterial cultures under different culture conditions. It has now been used to explore microbial communities and gene functions in samples from human (Lagier et al., 2018), animals (Wang et al., 2021), plants (Li et al., 2023) and environment (Sun et al., 2023). Metagenomic sequencing and culturomics are complementary approaches that can be used in combination to explore the true picture of microbial communities in a given sample (Ji et al., 2024). There are recent studies in which combined metagenomic sequencing and culture-dependent methods were employed to identify the causal pathogens and disease diagnosis in plants (Zhang et al., 2023; Yuan et al., 2023b).

At present, the MBB research mainly focuses on pathogen identification and patho-biology, preliminary exploration of disease mechanisms, and disease prevention and control (Huang et al., 2024; Baharuddin, 1997; Krawczyk and Łochyńska, 2020). However, as the global climate outlook and agricultural practices are changing, it is likely that new pathways for pathogen transmission may continue to emerge (Rosario et al., 2020). Given the complexity of sericulture and mulberry production systems, it becomes necessary to conduct more in-depth research on the causal pathogens of MBB and their transmission mechanism. It is said that, among non-human factors, meteorological factors play a key role in the spread of pathogens, as microorganisms, including bacteria, can exist stably in aerosols and can spread from place to place with atmospheric movement (Fröhlich-Nowoisky et al., 2016; Jung et al., 2018). Therefore, in this context, it is important to study the driving factors of MBB transmission, especially meteorological factors. Such studies are expected to help us understand the disease to a greater extent, and will certainly provide basis for the development and implementation of measures for the prevention and control of MBB.

In the present study, the MBB disease samples were collected from eight provinces in China including Guangdong, Jiangsu, Zhejiang, Sichuan, Shaanxi, Yunnan, Anhui, and Guangxi Zhuang Autonomous Region. The samples were analyzed for microbiome composition and diversity in the disease samples through combined metagenomic sequencing and culturomics methods. The suspected pathogens were screened out, and pathogenicity tests were performed on mulberry to verify the Koch’s postulates. The screened pathogenic strains were combined with their distribution patterns in the samples to determine the main causal pathogen of MBB. Then, the pathogens of MBB in combination with the local meteorological data (15 days before and after the sample collection) were analyzed to draw a potential relationship between each pathogen and relevant meteorological factors. Finally, the selected primary pathogens were re-inoculated into mulberry. Following exhibition of typical symptoms, mulberry plants were used for metagenomic sequencing to investigate the changes in the diversity of pathogens in the disease samples. The results of this study provide a reasonable basis for the focused research on the prevention and control of MBB, and will also contribute to the sustainable development of the global silk industry.

## 2 Materials and methods

**Figure 1:**
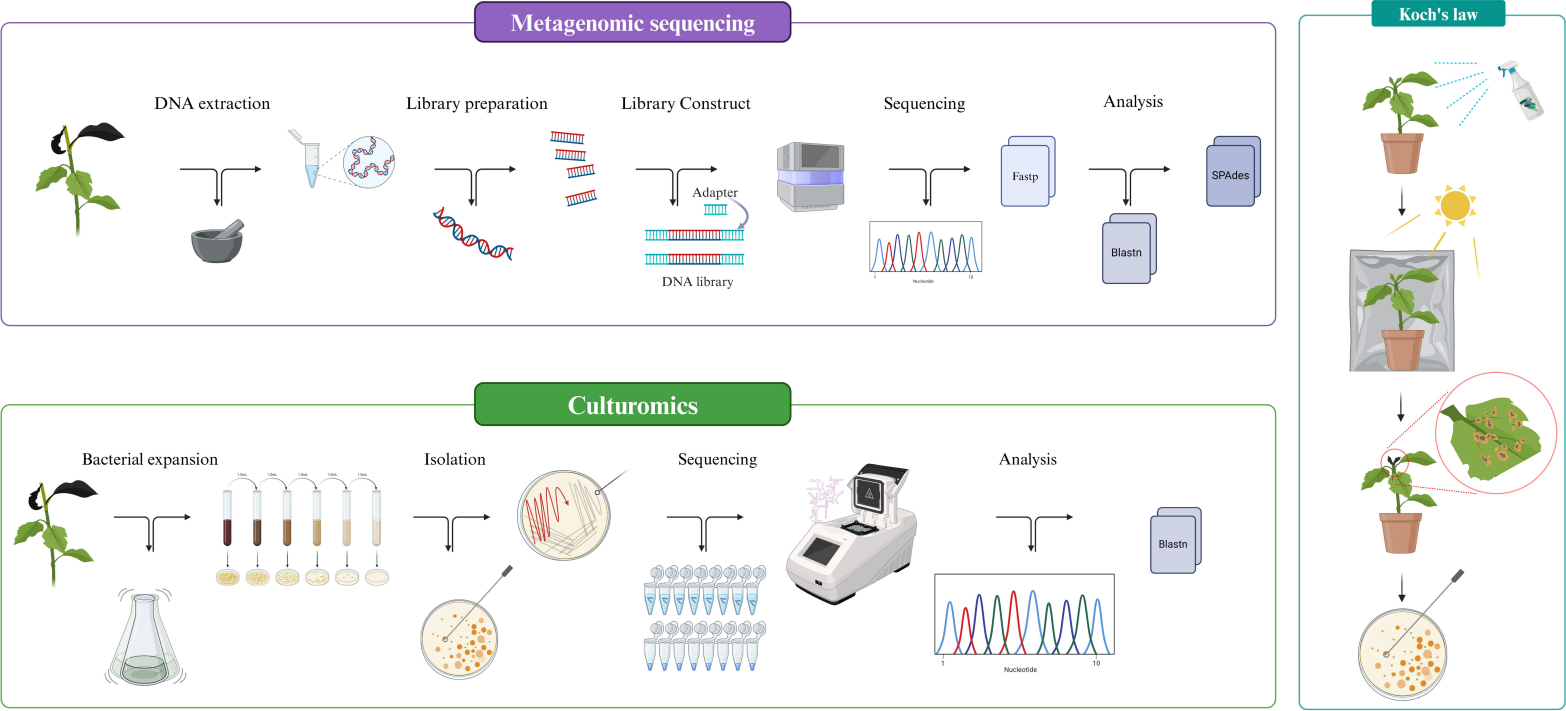
Experimental flowchart.

The entire experimental flow of this study is presented in the form of schematic as **Figure 1**.

### 2.1 Metagenomics-based detection of MBB field disease samples

#### 2.1.1 Locations and sample material

**Figure 2:**
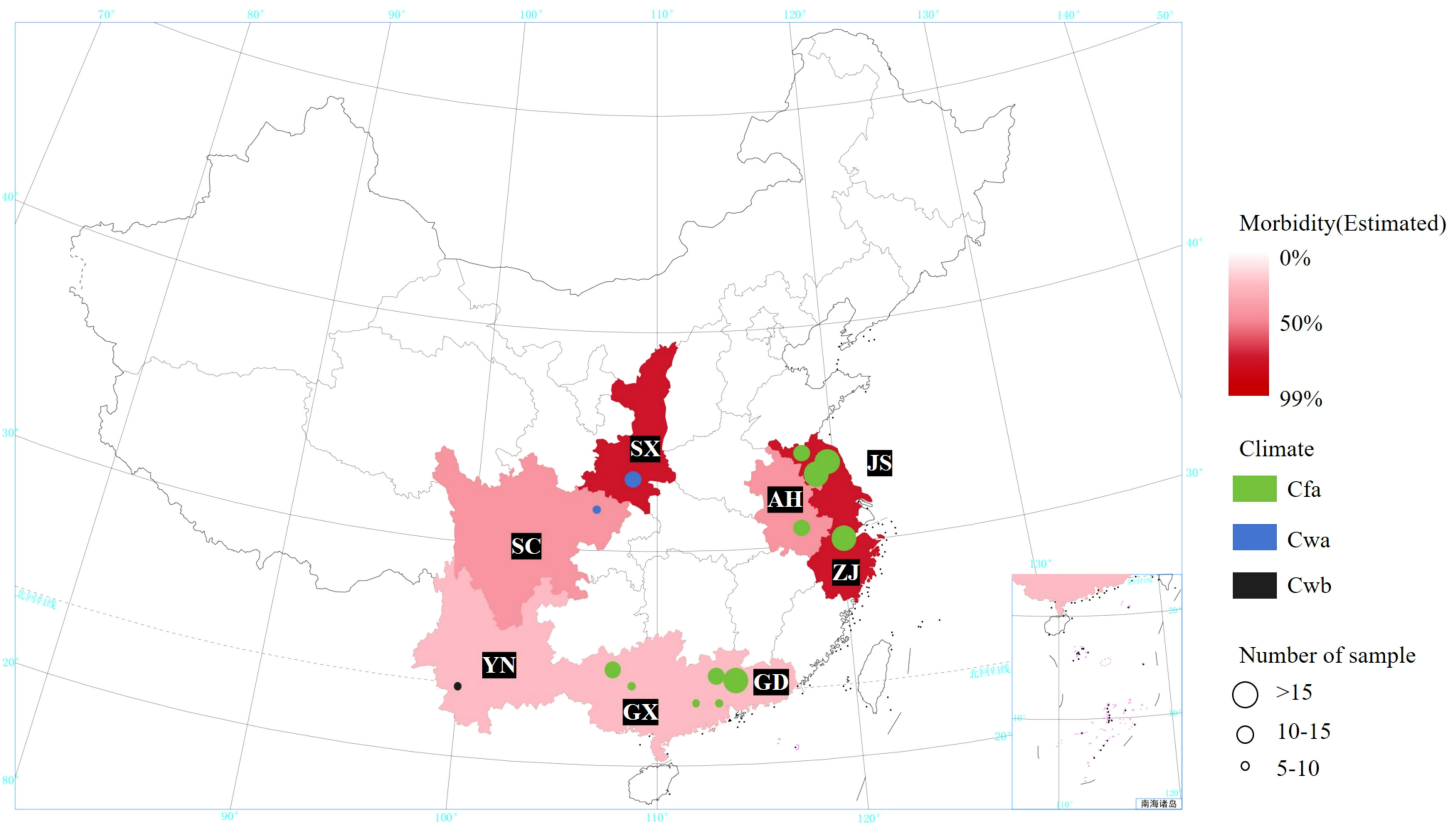
Information on Mulberry Bacterial Blight samples and collection locations. SX, Shaanxi province; SC, Sichuan province; YN, Yunnan province; GD, Guangdong province; ZJ, Zhejiang province; JS, Jiangsu province; AH, Anhui province; GX, Guangxi Zhuang autonomous region. Cfa, Humid subtropical climate; Cwa, Dry-winter humid subtropical climate; Cwb, Dry-winter subtropical highland climate.

The MBB samples were collected from 16 different locations in eight provinces of China. The selection of these regions was based on the fact that these provinces are sericulture intensives regions in China and reportedly contributed to 91.39% of the total sericulture production in 2023^1^. These provinces cover three different types of Köppen climate (see **Table 1**; **Figure 2**).

**Table 1:** Information of sample source and collection locations.

Sample code	Date	Location			TEMP (°C)	SSD (h)	PRCP (mm)	RH (%)	WDSP (km/h)	PRES (hPa)	K ö ppen climate	Mor* (%)	Season	Cultivar	Species	Symptom
		Region		Latitude and longitude												
X	2024.8.18	AH	Hefei	117.37°E,31.53°N	30.5	13.28	65.9	79.87	6.1	998.19	Cwa	16.67	summer	Qiang Sang NO.1	Morus multicaulis Perr.	terminal bud curl
ZA1-5	2024.3.11	GD	Yingde	113.41°E,24.18°N	18	12.01	10.9	77.18	5.8	1008.59	Cfa	6.67	spring	Yuesang11	*M. atropurpurea*	terminal bud black
ZA6	2024.3.13	GD	Zhongshan	113.46°E,22.41°N	20	12.02	16	78.76	9.5	1000.46	Cfa	6.67	spring	Sha2xLun109	*M. atropurpurea*	terminal bud black
ZC	2024.3.19	GD	Zengcheng	113.81°E,23.26°N	19.5	12.02	7	77.36	6.59	1012.55	Cfa	50.00	spring	Shengxiansang	M. multicaulis Perr.	terminal bud black
ZD	2024.3.19	GD	Zengcheng	113.81°E,23.26°N	19.5	12.02	7	77.36	6.59	1012.55	Cfa	63.33	spring	Da10	*M. atropurpurea*	terminal bud black
ZK	2024.3.19	GD	Zengcheng	113.81°E,23.26°N	19.5	12.02	7	77.36	6.59	1012.55	Cfa	50.00	spring	Kangqing10	*M. atropurpurea*	terminal bud black
ZL	2024.3.19	GD	Zengcheng	113.81°E,23.26°N	19.5	12.02	7	77.36	6.59	1012.55	Cfa	43.33	spring	Lunbai10	M. alba L.	terminal bud black
Y	2024.3.30	GD	Yunan	111.54°E,23.23°N	20.1	12.02	17.9	79.36	5.37	1007.29	Cfa	16.67	spring	Qiang Sang NO.1	M. multicaulis Perr.	terminal bud black
A	2023.10.15	GX	Huanjiang	108.26°E,24.83°N	21	12.83	1.2	70	3.1	985.95	Cfa	10.00	autumn	Gui Sang12	M. atropurpurea Roxb.	terminal bud black
W	2024.8.13	GX	Mashan	108.20°E,23.73°N	28.5	12.92	155.2	84.24	5.14	980.13	Cfa	6.67	summer	Guisang12	M. atropurpurea Roxb.	terminal bud black
DF	2023.5.19	JS	Dafeng	120.56°E,33.36°N	18	14.16	40.6	69	3.1	985.95	Cfa	73.33	summer	Qiang Sang NO.1	M. multicaulis Perr.	terminal bud black
HA	2023.5.29	JS	Haian	120.47°E,32.53°N	20	14.17	24	70	3.45	998.67	Cfa	96.67	summer	Qiang Sang NO.1	M. multicaulis Perr.	terminal bud black
S2	2024.6.11	JS	Dongtai	120.07°E,32.33°N	25	14.27	222.1	74.42	6.32	1006.27	Cfa	73.33	summer	Yu71-1	M. multicaulis Perr.	terminal bud black
S3	2024.6.11	JS	Sheyang	119.67°E,34.07°N	24	14.35	92.4	76.26	9.89	1006.59	Cfa	70.00	summer	Yu71-1	M. multicaulis Perr.	terminal bud black
S4	2024.6.11	JS	Sheyang	119.67°E,34.07°N	24	14.35	92.4	76.26	9.89	1006.59	Cfa	73.33	summer	Xuan792	M. multicaulis Perr.	terminal bud black
S5	2024.6.11	JS	Suining	114.80°E,34.18°N	27	14.37	59.5	60.3	7.23	1002.83	Cfa	73.33	summer	Qiang Sang NO.1	M. multicaulis Perr.	terminal bud black
S1	2024.6.9	JS	Haian	120.47°E,32.53°N	25	14.25	248.7	75.3	9.17	1005.91	Cfa	80.00	summer	Yu71-1	M. multicaulis Perr.	terminal bud black
S6	2024.8.14	JS	Sheyang	119.67°E,34.07°N	29.5	13.37	170.2	83.78	7.12	1004.96	Cfa	63.33	summer	Yu71-1	M. multicaulis Perr.	terminal bud black
R1-R2	2024.5.27	SC	Nanchong	105.86°E,30.74°N	23	13.71	45.4	74.45	6.41	969.38	Cwa	40.00	summer	Qiang Sang NO.1	M. multicaulis Perr.	terminal bud black
T1	2024.7.3	SX	Ankang	108.51°E,32.89°N	27.5	14.05	249	86.49	5.56	939.68	Cwa	60.00	summer	Nongsang14	M. multicaulis Perr.	terminal bud black
U	2024.7.25	YN	Puer	100.82°E,23.09°N	27.5	13.47	249.2	84.01	4.92	877.37	Cwb	10.00	summer	Qiang Sang NO.1	M. multicaulis Perr.	terminal bud black
P1-P8	2024.4.20	ZJ	Hangzhou	118.21°E,29.11°N	18.5	12.89	103.3	80.03	6.17	1006.55	Cfa	96.67	spring	Qiang Sang NO.1	M. multicaulis Perr.	terminal bud black

TEMP, Monthly average temperature; SSD, Sunshine duration; PRCP, Monthly total precipitation; RH, Relative humidity; WDSP, Wind direction speed; PRES, Station pressure; SX, Shaanxi province; SC, Sichuan province; YN, Yunnan province; GD, Guangdong province; ZJ, Zhejiang province; JS, Jiangsu province; AH, Anhui province; GX, Guangxi Zhuang autonomous region; Cfa, Humid subtropical climate; Cwa, Dry-winter humid subtropical climate; Cwb, Dry-winter subtropical highland climate; Mor., Morbidity%;

Accordingly, field investigations were extended to 16 diverse locations including Hangzhou City, Zhejiang (P1-P8); Hai’an City (S1, HA); Sheyang City (S3, S4, S6); Dongtai City (S2); Suining City (S5) and Dafeng (DF) City, Jiangsu; Hefei City, Anhui (X); Ankang City, Shaanxi (T1); Puer City, Yunnan (U); Nanchong City, Sichuan (R1-R2); Yingde City (ZA1-5); Yunfu City (Y); Zhongshan City (ZA6); Guangzhou City (ZC, ZD, ZK and ZL), Guangdong; Mashan City (W); Huanjiang, City (A), Guangxi. Based on the source location, the collected samples were divided into eight groups, and named as GX (W and A), GD (ZA6, ZC, ZD, ZK, ZL, ZA1-5 and Y), ZJ (P1-P8), SC (R1-R2), JS (DF, HA, S1, S2, S3, S4, S5 and S6), YN (U), AH (X), SX (T1).

**Figure 3:**
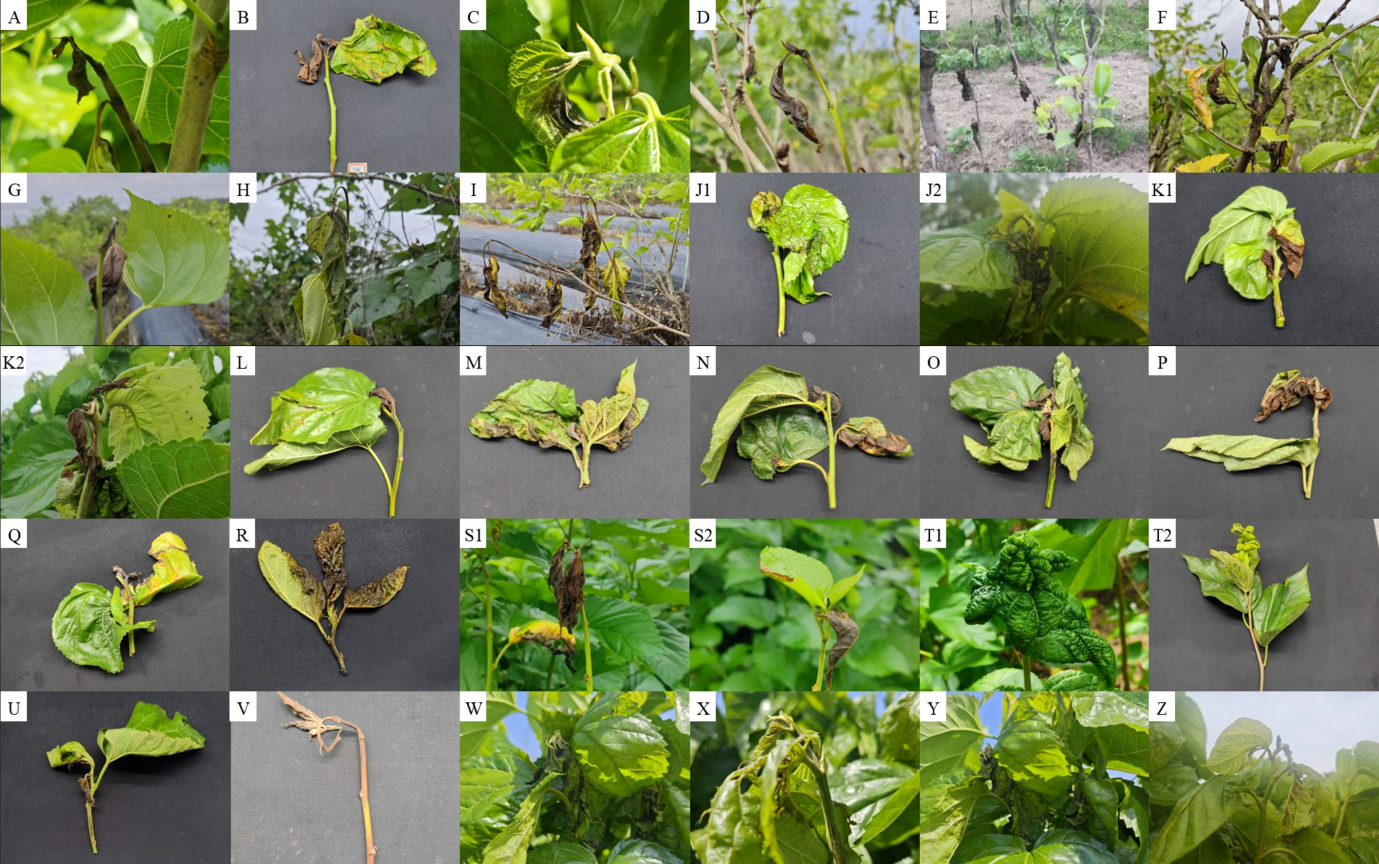
Samples of Mulberry bacterial blight collected from different locations. **(A)** Hai’an, Jiangsu sample (HA). **(B)** Dafeng, Jiangsu sample (DF). **(C)** Huanjiang, Guangxi sample (A). **(D)** Qingyuan, Guangdong sample (ZA1-5). **(E)** Zhongshan, Guangdong sample (ZA6). **(F–I)** Zengcheng, Guangdong sample (ZC). **(J1, J2)** Hangzhou, Zhejiang sample (P1-P8). **(K1, K2)** Nanchong, Sichuan sample (R1-R2). **(L)** Yancheng, Jiangsu sample (S1). **(M)** Nantong, Jiangsu sample. **(N)** Dongtai, Jiangsu sample (S2). **(O, P)** Sheyang, Jiangsu sample (S3, S4). **(Q)** Ankang, Shanxi sample (T1). **(R)** Puer, Yunnan sample (U). **(S1, S2)** Mashan, Guangxi sample (W). **(T1, T2)** Hefei, Anhui sample (X). **(U)**, Sheyang, Jiangsu sample (S6). **(V)** Yunan, Yunfu sample(Y). **(W)** Haining, Zhejiang sample (P1-P8). **(X)** Yancheng, Jiangsu sample (S5). **(Y)** Fuyang, Anhui sample (ZD). **(Z)** Hanyin Shanxi sample (ZL).

Detailed information of all samples and collection sites is shown in **Table 1**, and **Figures 2**, **3**. A five-point sampling method was used to randomly survey 30 mulberry trees in the local mulberry fields where samples were collected. The number of mulberry trees showing MBB symptoms and the number of healthy mulberry trees were counted to calculate the morbidity (**Table 1**). The diseased parts of mulberry trees were collected with ethanol-disinfected scissors, and placed into zip lock PE bags, and kept in a refrigerator at 4°C until used for the bacterial isolation. The collected MBB disease samples were washed with soapy water, disinfected with 75% ethanol, and then rinsed with sterile water for at least three times. Then the samples were frozen (-80°C) in sterile bags for further analysis. Climate type of the sampling sites were determined according to the Köppen climate classification (**Table 1**). At the same time, the meteorological data including Temperature (TEMP), Precipitation (PRCP), Relative humidity (RH), Wind direction speed (WDSP), Station pressure (STP) of all sample collection sites (15 days before and after the sampling period) were recorded (**Table 1**). Meteorological data was downloaded from the open data platform of the National Meteorological Information Center, China Meteorological Administration via its Application Programming Interface (http://data.cma.cn).

#### 2.1.2 Cultivation of mulberry for replanting

The mulberry tree variety Qiangsang No. 1 (*Morus alba* var. *multicaulis*) was used for grafting in this experiment. The plant material was sourced from the mulberry research station of the South China Agricultural University, China (113. 35°E, 23. 16°N).

### 2.2 Metagenomic sequencing

#### 2.2.1 DNA extraction, metagenomic sequencing, assembly, read quality control and processing

The samples (**Table 1**; MBB sample S1) collected from Jiangsu province exhibiting typical symptoms of the MBB disease were used for metagenomic sequencing. As per the instructions of the bacterial genomic DNA extraction kit (CW0552, CoWin Biosciences), the diseased-healthy junction area of the diseased sample was cut and used for total DNA extraction. The extracted total DNA was quality controlled by agarose gel electrophoresis, and the qualified DNA was stored at -20°C. The qualified DNA samples were randomly broken into fragments of about 350 bp in length using a Covaris ultrasonic crusher (Covaris, USA) to construct a small fragment genomic DNA library. The library construction process was carried out according to the instructions of the VAHTSTM Universal DNA Library Prep Kit for Illumina^®^ Library Construction Kit. Following library construction, quality control was performed using RT-qPCR and Agilent 2100 Bioanalyzer (Agilent Technologies, USA). The DNA library that passed the quality inspection was sequenced on the Illumina Novaseq6000 (Illumina, USA) high-throughput sequencing platform, using the sequencing strategy PE150 (Pair-End 150). To ensure the accuracy and reliability of subsequent analysis and test results, the sequencing data was subjected to further statistical screening. These checks included sequencing data volume, quality index, and GC ratio distribution. The data from the Illumina high-throughput sequencing platform was converted into raw reads using CASAVA software. Then the adapter sequences and low-quality sequences in the raw data were filtered to ensure the quality of the data analysis. The raw sequences were filtered to obtain high-quality clean reads. When the N content in any read exceeded 10% of the base number of the read, the paired reads were removed. When the number of low-quality (Q<=5) bases in any sequencing read exceeded 50% of the base number of the read, the paired reads were removed. Sequence assembly was performed using SPAdes v. 3. 5. 0 software (Bankevich et al., 2012).

#### 2.2.2 Annotation of metagenomic data

Using the BLASTn (v2. 5. 0+) alignment strategy (Zhang et al., 2000), sequencing tags were aligned against the bacterial and fungal databases in the National Center for Biotechnology Information (NCBI, http://ftp.ncbi.nlm.nih.gov/) nt database. Based on multiple dimensions such as sequence alignment similarity, alignment length, and sequence integrity, the optimal alignment results were selected through a similarity evaluation algorithm to annotate the tag sequences, and the microbial diversity in the samples was analyzed. The abundance of species was calculated based on the sequencing depth and assembly length of the sequence tags. The MEGAN software (Version 6. 20. 0) was used to visualize the microbial species and quantities (Huson et al., 2016). High-abundance microbial species in MBB samples were screened and preliminarily identified as pathogens.

#### 2.2.3 Screening and annotation of suspected pathogens

Based on the high-throughput sequencing data assembly and experimental verification results, the complete rRNA sequence was obtained. The rRNA sequence was annotated using blastn. Based on the rRNA sequences of closely related species in the nt database, the length and GC ratio of the 16S/18S, ITS1, 23S/28S, ITS2, and 5S intervals were counted respectively. With 250 bp as the statistical threshold, the GC ratio distribution of the sequence was counted and the GC ratio distribution map was drawn by sliding 10 bp each time. The rDNA sequences of species with high percent identity to the alignment results were downloaded from the NCBI for phylogenetic tree construction. MUSCLE v. 3. 8. 31 software (Edgar, 2004) was used for sequence alignment between multiple species. jModelTest2. 1. 7 was used to test the nucleic acid model of the selected sequence DNA (Posada, 2008). The substitution model with the smallest AIC (Akaike Information Criterion) value was taken as the optimal phylogenetic tree substitution model. The phylogenetic tree was constructed using the RAxML8. 1. 5 software (Stamatakis, 2014), using the ML method (maximum likelihood) with the bootstrap value set to 1000.

### 2.3 Culturomics

#### 2.3.1 Isolation and culture

As described in our previous publication Yuan et al. (2023a); the stems of mulberry plants with typical bacterial blight symptoms were selected (**Figure 3**; **Table 1**) and washed with soapy water and then rinsed with tap water for five min. Tissue blocks of approximately 0. 5 cm×0. 5 cm×0. 5 cm were cut at the junction of diseased and healthy portion. Blocks were disinfected with 75% ethanol for 30 s, soaked in 0. 1% mercuric chloride for five minutes, rinsed with sterile water for three times, and dried using sterile filter paper. After drying, tissue block were cut into pieces under sterile conditions and put into a conical bottle containing sterile saline in the constant temperature shaker (Shanghai bluepard instruments Co., ltd) at 28°C and 180 rpm, separating fluid sample at 2h and 12h. Three technical replicates were used for each mulberry sample. To increase the opportunity of isolating the diverse bacteria, a culturomics strategy was devised. This strategy included two different culture media (described below) used for the bacterial isolation. After shaking, samples were subjected to a 10 fold gradient dilution, and then 100μL aliquots from 1 × 10^4^ to 1 × 10^8^ gradient were taken and coated on the Luria Bertani (LB) agar medium (Peptone 10g·L^-1^, Yeast Extract 5g·L^-1^, NaCl 5g·L^-1^, D-Glucose 1g·L^-1^, Agar 18g·L^-1^, pH 7. 0) and Nutrient agar (NA) medium (Peptone 10g·L^-1^, Beef Extract 3g·L^-1^, NaCl 5g·L^-1^, Agar 18g·L^-1^, pH 7. 0) (Sarkar et al., 2022). At least three technical replicates were used for each gradient on different culture media.

#### 2.3.2 Purification and cryopreservation of bacterial cultures

As previously described by Singh et al., 2017, colonies were picked from primary isolation media once every two days, for eight days, and subcultured onto new plates. All isolated strains were subcultured in the LB agar medium until they grew well. Colony morphology, texture, color and consistency were comprehensively assessed to ensure that only pure cultures of a single bacterial species were present on each medium. Pure cultures were stored on plates and agar slants for short periods, and colonies were also placed in sterile 30% glycerol and stored at −80°C for future use (Vanneste et al., 2013).

#### 2.3.3 Bacterial 16S rRNA identification

Nucleic acids were extracted from pure cultures of the bacterial strains using a modified CTAB method. The *16S rDNA* was then amplified by PCR using bacterial 16S universal primers 27F (5’-AGAGTTTGATCCTGGCTCAG-3’) and 1492R (5’-GGTTACCTTGTTACGACTT-3’) (Weisburg et al., 1991). The PCR reaction system (25 μL) comprised of 2x Taq MIX 12. 5 μL (Genesand Biotech Co., Ltd), forward primer 1 μL, reverse primer1 μL, template DNA 1 μL, and dd H_2_O. The PCR (Takara Bio Inc. Japan) reaction conditions were as follows: denaturation at 94°C for 5 min, followed by 33 cycles of: denaturation at 94°C for 30 s, annealing at 55°C for 30 s, extension at 72°C for 90 s, final extension at 72°C for 10 min. The PCR amplification products were subjected to analysis via 1. 2% agarose gel electrophoresis to confirm the size of the target amplicons. After gel extraction, the fragments were purified and sent to Sangon Biotech (Shanghai) Co., Ltd. for bidirectional sequencing. The obtained *16S rRNA* gene sequences were then assembled and submitted to the NCBI for BLAST analysis to facilitate identification of bacterial species (Ye et al., 2006). Phylogenetic trees were constructed using ML (maximum likelihood) method (MEGA 11 software) (Shen et al., 2002). Finally, based on the BLAST alignment results, strains with percent identity between 95% and 97%, query cover between 95% and 100%, were preliminarily identified at the genus level, while those with percent identity between 97% and 100%, query cover between 99% and 100%, were preliminarily identified at the species level (Gilbert et al., 2010).

### 2.4 Pathogenicity testing of pathogenic bacteria

As described by Chen et al. (2021), the species or genus of each isolate were initially identified based on the *16s rRNA* comparison results. Bacterial colony composition of samples were identified using metagenomic and culturomics results. Bacteria that co-appeared in the results of the two identification methods were initially screened and tentatively designated as suspected pathogenic bacteria. In addition, the pathogens of several common plant diseases were screened out based on existing literature reports (Luo et al., 2022; Yuan et al., 2023a; Popović et al., 2021). The bacteria were selected according to the principle of taking at least two strains from each different sample as biological replicates, and the pathogenicity test was performed. Briefly, pure cultures of all suspected pathogenic bacteria were incubated overnight in the LB liquid culture medium, centrifuged at 5000 rpm for three min to collect the bacterial cells, then diluted with sterile water to 1×10^7^.

Healthy mulberry plants were taken and the inoculation site was selected at the surface of the branch between 2-3 true leaves below the top bud. The selected inoculation site was disinfected with 75% alcohol, then 500 μL of bacterial suspension was slowly injected into the plant tissue with a sterile syringe, and inoculation site was wrapped the with sterile gauze. The samples were placed in a constant temperature and light incubator at 28°C, RH 85-95%, and 12h: 12h light cycle. The sterile water injection was used as the control (CK) group. Three technical replicates were used for each strain.

The disease situation was observed and the disease index (Di) was calculated after three days (Krawczyk and Łochyńska, 2020). The Di was calculated as follows:


$$D_{i}=\sum \frac{D_{l}*N_{d}}{N_{t}*N_{s}}$$

Di: Disease Index

Dl: Disease Level

Nd: number of disease tender leaves

Nt: number of total tender leaves

Ns: number of biological replicates

After the bacteria were re-inoculated, the samples that showed symptoms of disease were isolated and cultured to observe whether the bacteria inoculated in the sample could be re-isolated. The re-isolated bacteria were molecularly identified based on 16s rDNA, as mentioned in 2. 3. 1 to 2. 3. 3 of materials and methods. For clarity. the pathogens isolated/identified through the pathogenicity test were named as the "isolated" group. The phylogenetic tree including all pathogens was constructed using ML method (MEGA11 software).

### 2.5 Metagenomic sequencing of re-inoculated samples

Upon exhibition of typical symptoms, samples from two experimental groups were collected, including re-inoculated samples with *P. syringae* strain AHDX and re-inoculated samples with *P. syringae* strain ZJDX. The samples re-inoculated with *P. syringae* strain AHDX were designated as AHHG, and those re-inoculated with *P. syringae* strain ZJDX were designated as ZJHG. One group in which sterile water was inoculated to mulberry samples was designated as control (CK). For each group, three technical replicates were used, totaling nine samples. Metagenomic sequencing was performed to observe the microbial composition of the samples. The steps were essentially the same as described in 2. 2.

### 2.6 Correlation analysis between pathogenic bacteria and meteorological factors

As per the method of Rosario et al. (2020), Shapiro Wilk’s test was applied to evaluate the normality of the data. The data on bacterial isolation showed non-normal distribution, so the relationship between meteorological factors and bacteria was studied using the Spearman rank correlation test.

### 2.7 Data statistics, processing, and visualization

The data were prepared via Microsoft Excel 2019 (Microsoft, USA). The normalized abundances of bacteria at the species-level were analyzed via SPSS software (v21. 0), and the results were visualized with Origin 2024b. Heatmap visualization of the pathogenic bacteria and meteorological data was performed via the “heatmap” package in R software (v4. 3. 2). Alpha diversity was calculated using the picante package, PcoA analysis and sample significance tests were conducted using the vegan package, the Welch’s t-test was employed for T-tests. PLS-DA analysis was performed using the mixOmics package, and ggpubr was used for plotting. Phylogenetic tree construction was performed using MEGA11 and iqtree2 software, using the ML method. The refinement of the evolutionary tree was completed using the iTol online (https://itol.embl.de/) website (Letunic and Bork, 2016).

## 3 Results

### 3.1 Metagenomic sequencing results of MBB samples

#### 3.1.1 Sequencing results and quality control checks

The metagenomic sequencing yielded raw data of 4737133500 bp sequence, 31580890 reads, with a GC content of 52. 55%. Q30 and Q20 were both greater than 90%, indicating that sequencing results were of good quality and reliable. After quality control, clean data of 4477161900 bp was obtained, with a total of 29847746 reads. The GC content was 52. 72%, and Q20 and Q30 were both greater than 90%. To eliminate the influence of the host genome, mulberry genome GCA_012066045. 3 and OP161261. 1 were used as host controls (Jiao et al., 2020). After removing the influence of host genomes from clean data, a total of 6312994 reads were removed, accounting for 21. 15% of the total clean data. After the above quality control, 23534752 reads were finally obtained, including the longest fragment of 284255 bp, the shortest fragment of 56 bp, an average fragment length of 459bp, N50 345, L50 1727, and a GC content of 52. 48% (**Supplementary Table 1**).

#### 3.1.2 Sequence tag annotation, diversity analysis, and screening of suspected pathogens

**Figure 4:**
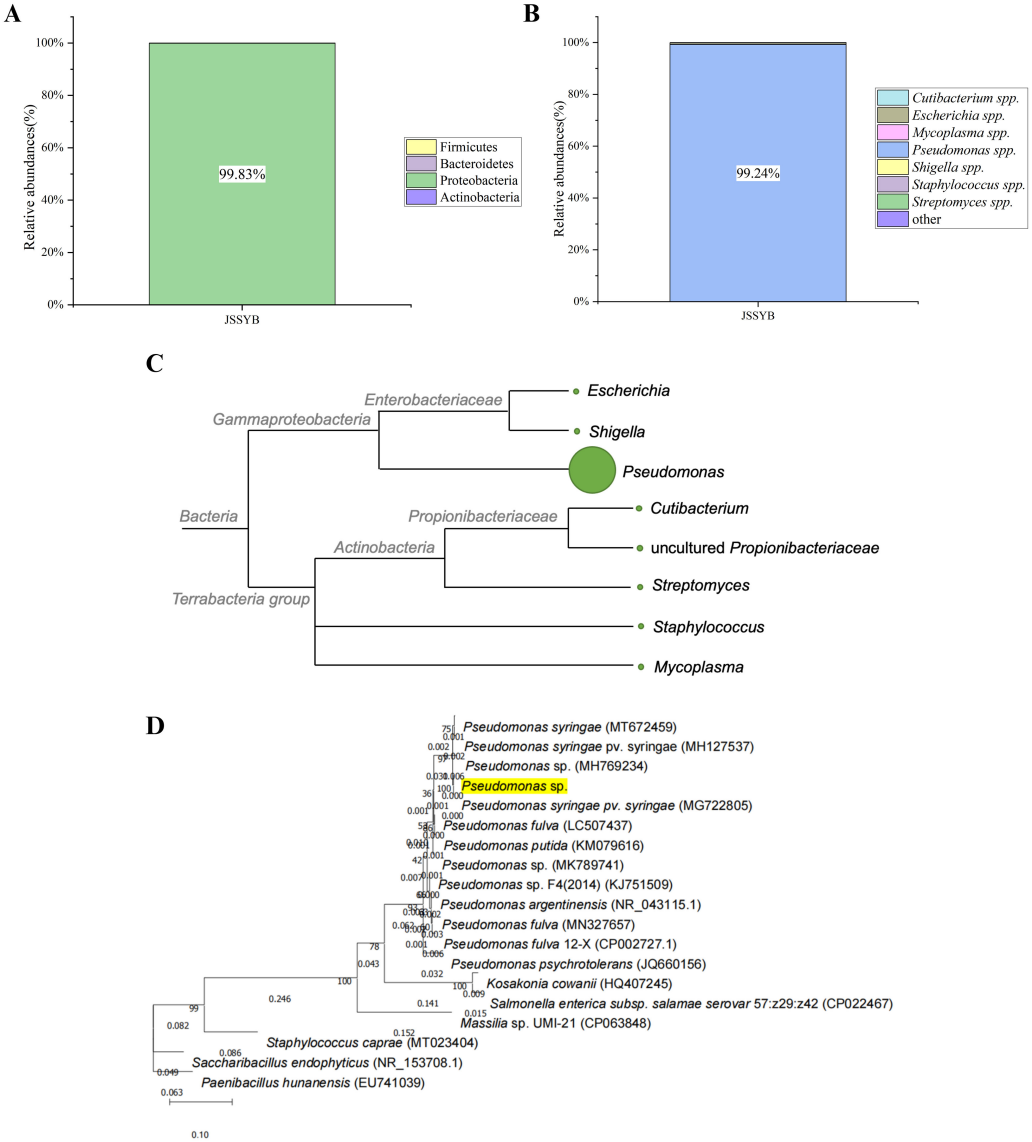
Metagenomic sequencing results of mulberry bacterial blight sample S1. **(A)** Relative abundance of bacteria at the phylum level. **(B)** Relative Abundance of Bacteria at the genus Level. **(C)** Cluster tree representing genetic relationships, with circle size indicating relative abundance of the genus. **(D)** Phylogenetic Tree based on rRNA (5S, 16S, 28S).

After annotation, it was observed that the microbial composition of the samples was at the phylum and genus levels. The relative abundance of Proteobacteria was the highest at the phylum level (99. 83%) (**Figure 4A**), and *Pseudomonas* spp. was the highest at the genus level (99. 24%) (**Figure 4B**). Based on the analysis of microbial species and quantity, combined with the examination of symptoms and relevant information, it was preliminarily determined that the suspected causal pathogen of MBB was *Pseudomonas* spp. (**Figure 4C**). The complete ribosome sequence of the suspected pathogenic bacteria was assembled from metagenomic data, and the total length of the ribosome sequence of the *Pseudomonas* spp. was approximately 5251bp, with a GC ratio of 52. 24% (**Supplementary Table 2**). The length information of the 16S/18S, ITS1, 23S/28S, ITS2, 5S/5. 8 intervals is shown in the **Supplementary Figure 1**. Based on *16Sr RNA* sequence alignment and construction of evolutionary tree, it was found that it clustered with *P. syringae*, suggesting that the suspected pathogens were all *P. syringae* (**Figure 4D**).

### 3.2 Culturomics results

#### 3.2.1 Bacterial isolation

A total of 103 samples were collected from 26 different times, locations or species in 20 different areas of eight provinces, and a total of 498 bacterial strains were isolated by culturomics. They were distributed in four phyla, six classes, 11 orders, 18 families and 48 genera (**Table 2**). At the phylum level, Proteobacteria was the most dominant community in all samples (410/502), and *Pseudomonas* spp. was the most frequently isolated genus at the genus level (138/410). Firmicutes was the second most dominant bacterial group, and at the genus level, *Paenibacillus* spp. showed the highest isolation frequency of 33/52. A total of 23 bacterial strains were isolated from *Actinobacteria* spp. *Curtobacterium* spp. and *Microbacterium* spp. were the two genera with the highest isolation frequency of 4/23, both of which were from Microbacteriaceae. In addition, we also isolated 13 strains of Bacteroidetes, with *Flavobacterium* spp. being the most abundant genus (9/13) (**Table 2**).

**Table 2:** Cumulative list of taxonomic information of culturable bacteria from mulberry bacterial blight samples.

Phyla	Classes	Orders	Families	General
Actinobacteria (23)	Actinobacteridae (23)	Actinomycetales (23)	Microbacteriaceae (19)	Cellulomonas *spp.* (2)
				Curtobacterium *spp.* (4)
				Gottfriedia sp. (1)
				Leucobacter *spp.* (2)
				Mammaliicoccus *spp.* (2)
				Microbacterium spp. (4)
				Niallia *sp.* (1)
				Priestia *spp.* (3)
			Micrococcaceae (1)	Brevibacterium *sp.* (1)
			Nocardioidaceae (1)	Nocardioides *sp.* (1)
			Propionibacteriaceae (2)	Luteococcus spp. (2)
Bacteroidetes (13)	Flavobacteria (13)	Flavobacteriia (12)	Flavobacteriaceae (12)	Chryseobacterium *spp.* (3)
				Flavobacterium *spp.* (9)
		Rhodobacterales (1)	Alcaligenaceae (1)	Atlantibacter *sp.* (1)
Firmicutes (52)	Bacilli (52)	Bacillales (50)	Bacillaceae (15)	Bacillus *spp.* (11)
				Exiguobacterium *sp.* (1)
				Saccharibacillus *spp.* (3)
			Paenibacillaceae (34)	Paenibacillus *spp.* (33)
				Paenimyroides sp. (1)
			Staphylococcaceae (1)	Staphylococcus *sp.* (1)
		Lactobacillales (2)	Streptococcaceae (2)	Lactococcus sp. (1)
				Aerococcus *sp.* (1)
Proteobacteria (410)	Alphaproteobacteria (3)	Rhizobiales (3)	Methylobacteriaceae (1)	Methylorubrum *sp.* (1)
			Rhizobiaceae (2)	Agrobacterium *spp.* (2)
	Betaproteobacteria (10)	Burkholderiales (10)	Comamonadaceae (10)	Comamonas *spp.* (7)
				Paracidovorax *spp.* (3)
	Gammaproteobacteria (397)	Enterobacterales (233)	Enterobacteriaceae (233)	Buttiauxella *spp.* (6)
				Citrobacter *spp.* (10)
				Cnuibacter *sp.* (1)
				Enterobacter *spp.* (35)
				Erwinia *spp.* (6)
				Escherichia *spp.* (4)
				Klebsiella *spp.* (7)
				Kosakonia *spp.* (43)
				Kluyvera *spp.* (13)
				Leclercia *spp.* (8)
				Lelliottia *spp.* (3)
				Pantoea *spp.* (74)
				Pectobacterium *spp.* (11)
				Pseudescherichia *spp.* (2)
				Rahnella *spp.* (9)
				Yersinia sp. (1)
		Pseudomonadales (143)	Moraxellaceae (5)	Acinetobacter *spp.* (5)
			Pseudomonadaceae (138)	Pseudomonas *spp.* (138)
		Sphingomonadales (2)	Sphingomonadaceae (2)	Sphingomonas spp. (2)
		Xanthomonadales (19)	Xanthomonadaceae (19)	Pseudoxanthomonas *spp.* (2)
				Stenotrophomonas *spp.* (15)
				Xanthomonas *spp.* (2)

The numbers within parentheses indicate the quantity of culturable bacteria strains belonging to this classification.

#### 3.2.2 Assessment of climate type of sample site and sample alpha diversity

We used the Köppen climate classification to estimate the climate types of the eight sample source regions. There were four groups (GX, GD, ZJ, and JS) of Cfa type, three groups (SC, SX, and AH) of Cwa type, and one group (YN) of Cwb type. As for the alpha diversity, it was found that the Shannon index of the Cfa type climate sample community was greater than that of the Cwa and Cwb types, while the Simpsen index of Cfa was greater than that of Cwb and Cwa types (**Table 3**; **Figure 2**).

**Table 3:** Diversity profile of the Mulberry Bacterial Blight wilt communities in mulberry.

	Province/Location								Climate type		
	GX	GD	ZJ	SC	JS	YN	AH	SX	Cfa	Cwa	Cwb
Number of isolates	32	106	178	25	104	24	17	12	420	54	24
Number of genera	12	19	20	4	10	9	17	3	41	17	10
Shannon	3. 19	3. 30	3. 81	1. 62	2. 08	2. 66	3. 38	1. 19	2. 71	2. 06	1. 84
Simpson	0. 89	0. 87	0. 90	0. 65	0. 70	0. 83	0. 94	0. 53	0. 88	0. 80	0. 83
Pielou’s evenness	0. 89	0. 78	0. 88	0. 81	0. 63	0. 84	0. 94	0. 75	0. 73	0. 74	0. 84
Berger-Parker	4. 00	4. 82	4. 34	2. 08	2. 60	3. 00	4. 25	1. 50	3. 89	2. 45	3. 00

SX, Shaanxi province; SC, Sichuan province; YN, Yunnan province; GD, Guangdong province; ZJ, Zhejiang province; JS, Jiangsu province; AH, Anhui province; GX, Guangxi Zhuang autonomous region; Cfa, Humid subtropical climate; Cwa, Dry-winter humid subtropical climate; Cwb, Dry-winter subtropical highland climate.

#### 3.2.3 Bacterial community composition of samples

**Figure 5:**
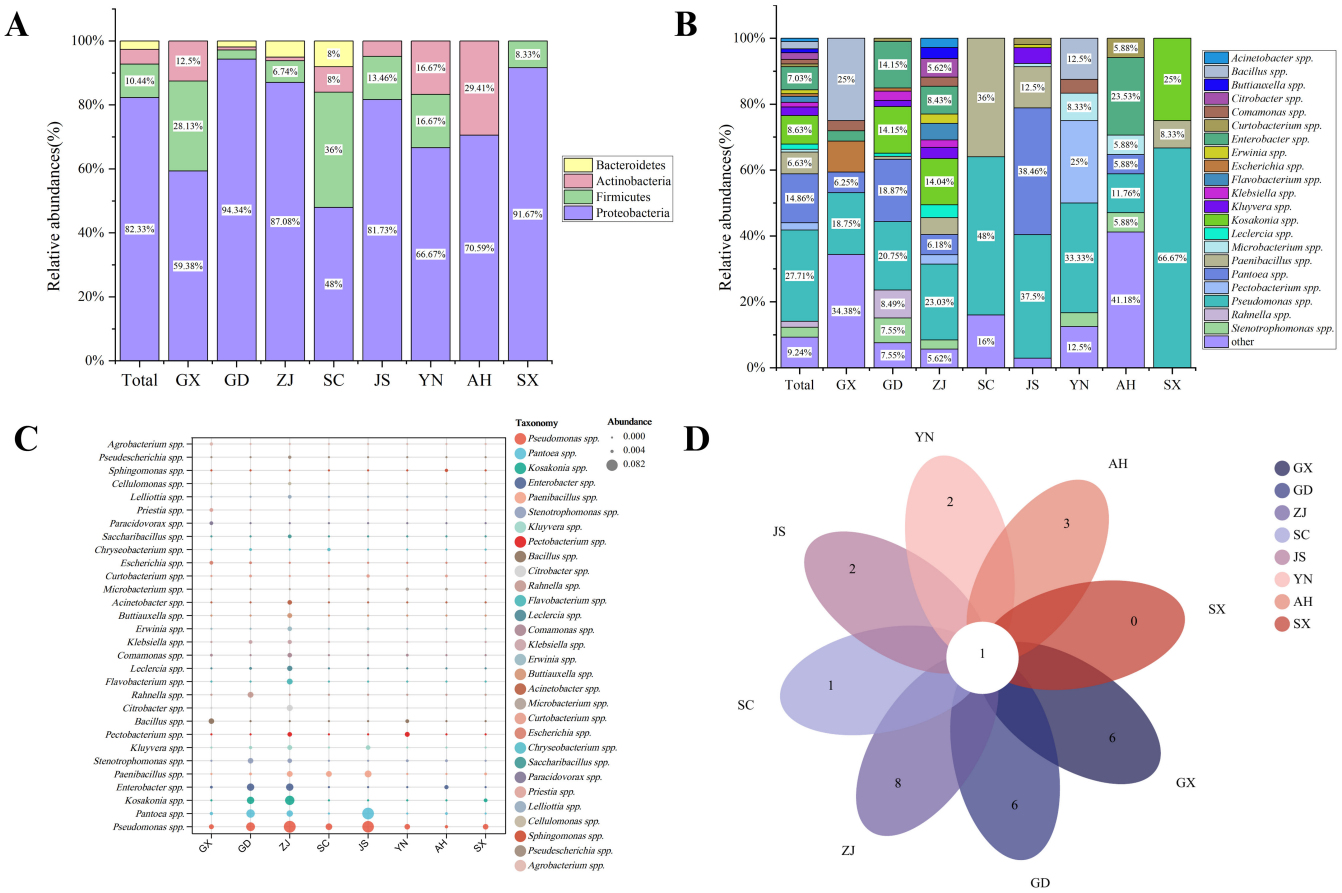
Results of bacterial communities isolated by culturomics. **(A)** Culturomics analysis of microbial composition of mulberry bacterial blight samples at the phylum level. **(B)** Culturomics analysis of microbial composition of mulberry bacterial blight samples at the genus levels. **(C)** Distribution of isolated bacteria across samples from eight different locations. **(D)** Venn analysis of the common and unique bacteria in samples from eight different locations.

The bacterial community composition of eight samples was calculated at the phylum and genus levels. At the phylum level, Proteobacteria was the most dominant community in each group (**Figure 5A**). Except for the GX and AH samples, *Pseudomonas* spp. was the most frequently identified genus in other samples. The most frequently identified genus in GX samples was *Bacillus* spp. (8/32; 25.00%), and the most frequently identified genus in AH samples was *Enterobacter* spp. (4/17; 23.53%) (**Figure 5B**). Overall, *Pseudomona* spp., *Pantoea* spp., and *Enterobacter* spp. were the most frequently identified genera in each group, and *Pseudomona* spp. co-appeared in eight groups (**Figure 5D**). The relative abundance bubble chart also showed that *Pseudomonas* spp., *Pantoea* spp., and *Enterobacter* spp. are widely present in each group (**Figure 5C**). At the family level, except for *Pseudomonas* spp. from Pseudomonadales, the other three genera were all from Enterobacterales (**Table 2**).

#### 3.2.4 Screening and distribution of suspected pathogens

**Figure 6:**
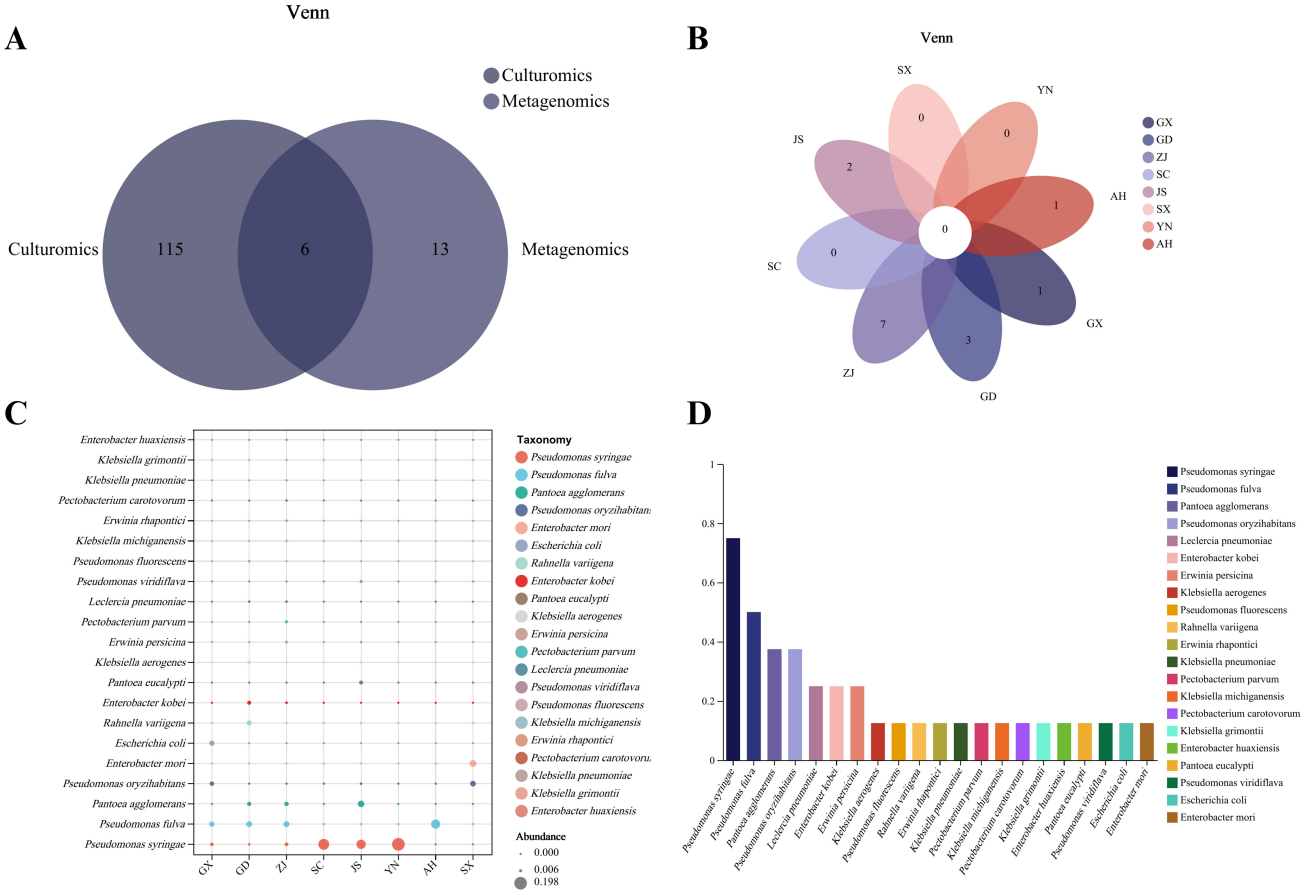
Results of metagenomic sequencing and culturomics. **(A)** Venn analysis of metagenomic sequencing and culturomics isolation and identification results. **(B)** Venn analysis of the presence of suspected pathogens in various isolated samples. **(C)** Relative abundance distribution of suspected pathogens in each sample. **(D)** Isolation rate of suspected pathogens in each sample.

To further screen the MBB pathogenic bacteria, we compared the culture group isolation and identification results with the metagenomic sequencing results at the species level. Six common bacterial species, including *P. syringae, P. fulva, P. oryzihabitans, P. fluorescens, P. viridiflava, and E. coli* were found to be common (**Figure 6A**). In this experiment, based on comprehensive consideration about the isolation frequency, relative abundance and previously reported genera/species of pathogenic bacteria, we screened 109 strains of suspected pathogenic bacteria, including 27 species from 12 genera, and analyzed their distribution in each group. We found that there were no species that appeared in common in the eight groups (**Figure 6B**). The top three most widespread species appearing in all groups were *P. syringae, P. fulva* and *P. agglomerans* (**Figures 6C, D**).

#### 3.2.5 Pathogenicity tests of suspected pathogens

**Figure 7:**
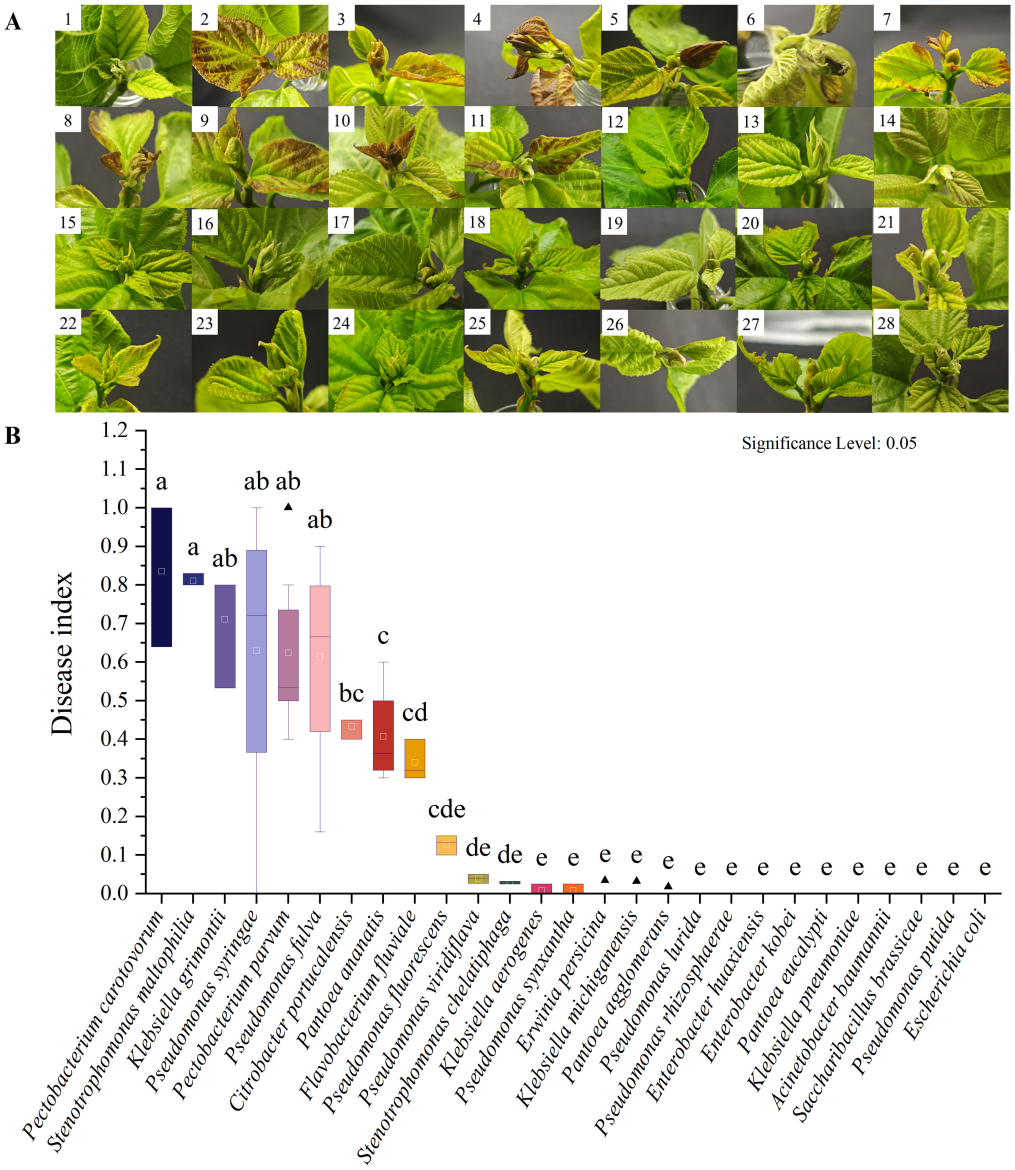
Results of pathogen verification by pathogenicity test. **(A)** Pathogenicity test results for 27 suspected pathogens. 1: CK. 2: *P. syringae* R2-2. 3: *P. fulva* P1-2-1. 4: *P. carotovorum* P7-24-3. 5: *P. fluorescens* ZA4-12. 6: *P. parvum* P7-2-5. 7: *F. fluviale* P5-24-1. 8: *C. portucalensis* P7-24-9. i: *K. grimontii* P7-24-8. 10: *S. maltophilia* P4-2-9. 11: *P. ananatis* ZA1-2. 12: *Pseudomonas lurida* P4-2-4. 13: *Pseudomonas rhizosphaerae* P2-24-16. 14: *Enterobacter huaxiensis* P7-2-2. 15: *P. agglomerans* ZA2-2. 16: *Klebsiella aerogenes* ZA4-8. 17: *Enterobacter kobei* ZK11. 18: *Pseudomonas synxantha* P4-24-1. 19: *Pantoea eucalypti* S1-1. 20: *Erwinia persicina* S1-10. 21: *P. viridiflava* S2-16. 22: *Klebsiella pneumoniae* P8-24-10. 23: *Acinetobacter baumannii* P8-24-12. 24: Saccharibacillus brassicae P8-24-13. 25: *Pseudomonas putida* P3-24-9. 26: *K. michiganensis* P3-24-13. 27: *Stenotrophomonas chelatiphaga* P4-2-10. 28: *E. coli* ZD6. **(B)** Disease index results for 27 suspected pathogens.

**Figure 8:**
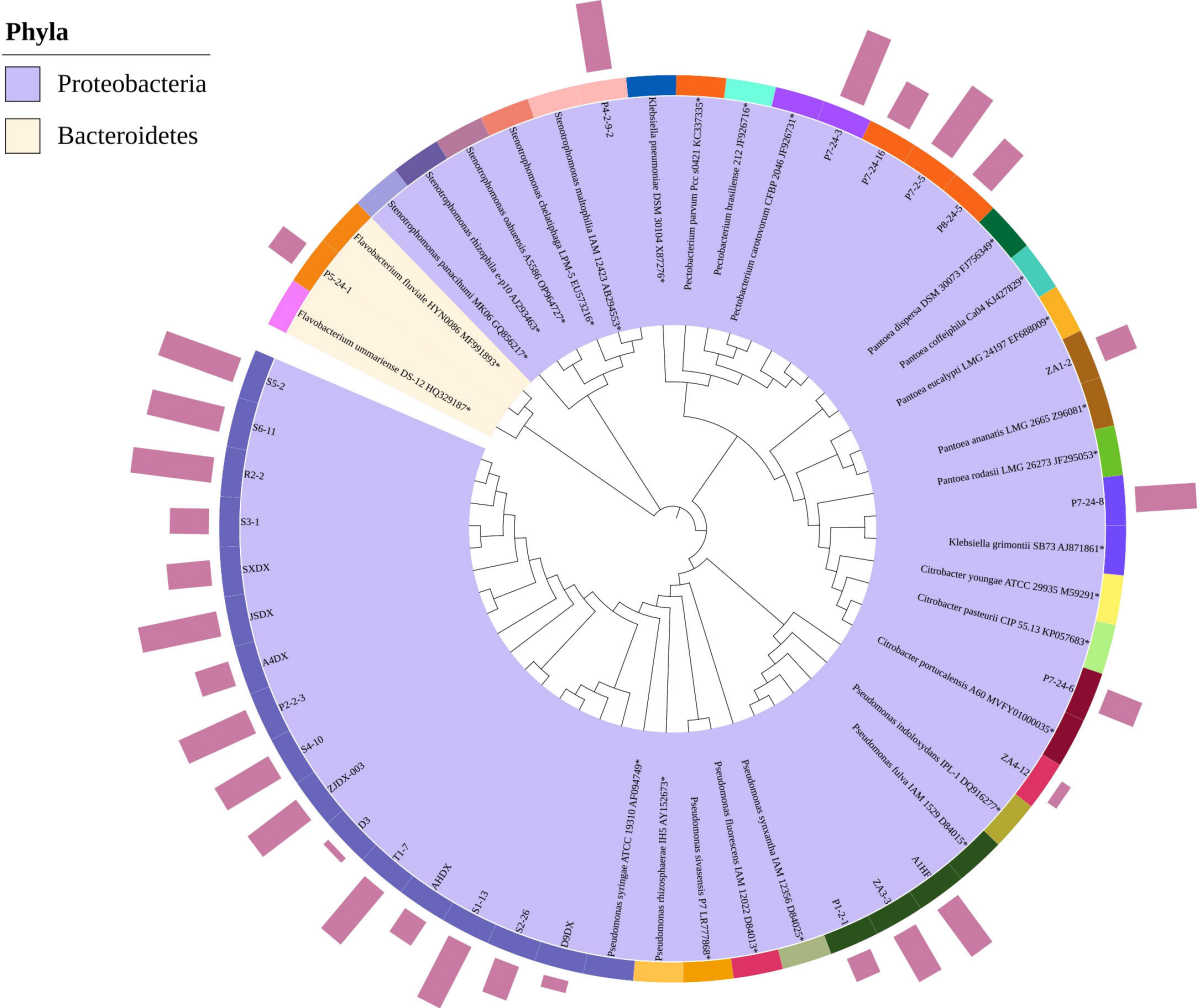
Phylogenetic tree of 10 causal pathogenic bacteria of mulberry bacterial blight. The inner circle represents the branching topology of the phylogenetic tree, and the second circle indicates the strain numbers. *Represents the type strain of this species. The color in the second circle denotes the phylum-level classification of the strain, the third circle represents the species-level classification, and the bar chart in the fourth circle indicates the pathogenicity of the strain as tested in this study.

To further verify the pathogenicity of all suspected pathogens, we conducted pathogenicity tests on Qiangsang No. 1 mulberry to verify Koch’s postulates. The pathogenicity test results showed that from 27 suspected pathogenic bacterial species tested, the disease index of 10 species was significantly higher than that of the blank control (*P*<0. 05). These species included *P. syringae, P. fulva, P. fluorescens, Pectobacterium carotovorum, P. parvum, Stenotrophomonas maltophilia, K. grimontii, C. portucalensis, P. ananatis*, and *F. fluviale*, (**Figure 7A**). It was found that *P. carotovorum* was the most virulent strain (**Figure 7B**). The phylogenetic tree of the 10 pathogenic bacteria was constructed. They were found to be distributed in two Phyla (Bacteroidetes, Proteobacteria) and classified into the same species as the respective pathogens of the re-inoculated groups. (**Figure 8**).

#### 3.2.6 Distribution of pathogens, correlation analysis between meteorological factors and pathogens

**Figure 9:**
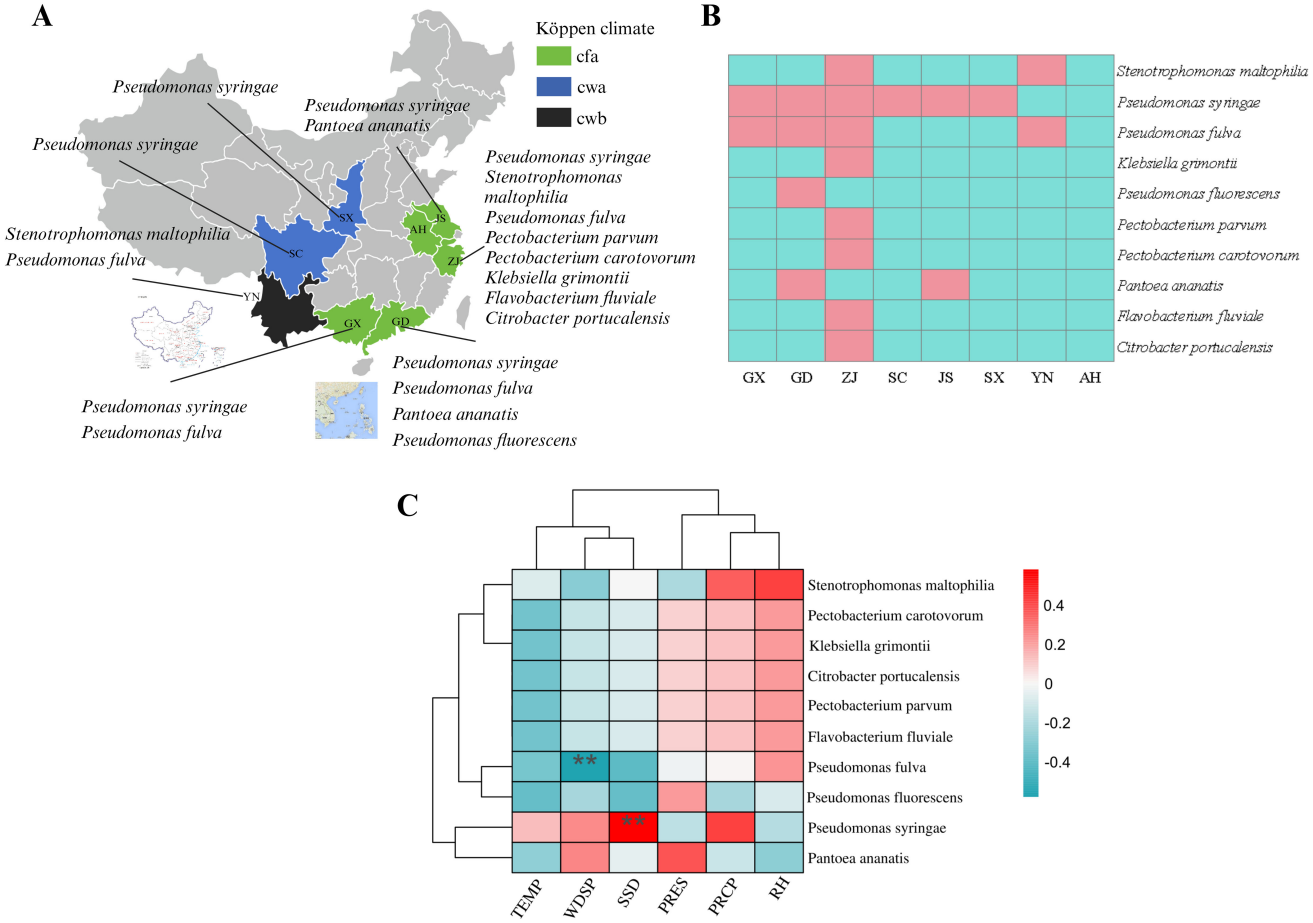
Distribution of pathogen and correlation analysis between meteorological factors and pathogens. **(A)** Pathogen distribution in different locations. Cfa, Humid subtropical climate; Cwa, Dry-winter humid subtropical climate; Cwb, Dry-winter subtropical highland climate. **(B)** Heatmap of distribution of 10 pathogenic bacteria in the sample. Red: existence in the sample, blue: non-existent in the sample. **(C)** Heatmap of pathogenic bacteria and local environmental factors. TEMP, Monthly average temperature; SSD, Sunshine duration; PRCP, Monthly total precipitation; RH, Relative humidity; WDSP, Wind direction speed; PRES, Station pressure. Red:, positively correlated, blue: negative correlation. **highly significant correlation (*P*<0. 01).

In order to analyze whether these 10 pathogens were widely distributed, the distribution of 10 pathogens in samples from the eight provinces was statistically analyzed. Briefly, *P. syringae* was isolated from samples from six provinces with a frequency of 75% (6/8). *P. fulva* was isolated from samples from four provinces with a frequency of 50% (4/8). *P. ananatis* and *S. maltophilia* were isolated from samples from two provinces with a frequency of 25% (2/8). *P. fluorescens, P. parvum, F. fluviale, C. portucalensis, K. grimontii* and *P. carotovorum* were only isolated from samples from one province, with a frequency of 12.5% (1/8) *F. fluviale*, *C. portucalensis*, and *K. grimentii* were only distributed in the cwa climate region, and only *P. syringae* was found in the cwb climate region (**Figures 9A, B**).

In this analysis, the distribution result of the above 10 bacteria in samples from different regions was combined with the local meteorological data to calculate the Spearman rank correlation test. As shown in the heatmap (**Figure 9C**), the occurrence of *P. syringae* was positively correlated with PRCP, WDSP, and TEMP, and was extremely significantly positively correlated with SSD (*P*<0. 01). The occurrence of *P. fulva* was only positively correlated with RH, not correlated with PRES and PRCP, negatively correlated with TEMP and SSD, and significantly negatively correlated with WDSP. *P. fluorescens* was positively correlated (*P*>0. 05) with PRES, and had no correlation or negative correlation (*P*>0. 05) with the other environmental factors. The occurrence of *P. ananatis* was positively correlated with PRES and WDSP, and negatively correlated with RH, PRCP, and TEMP. The occurrence of *P. parvum, F. fluviale, C. portucalensis, K. grimontii, P. carotovorum, S. maltophilia* was positively correlated with RH and PRCP (**Figure 9C**).

### 3.3 Results of metagenomic sequencing of re-inoculated samples

The read numbers in the raw data were between 13247304 and 32796380, and the base numbers were between 1987095600 and 4919457000 bp. After quality control, clean data was obtained, with read numbers between 12769796-31651202 and base numbers between 1915469400 and 4747680300bp. The Q30 and Q20 of all samples were greater than 90%, indicating good sequencing quality. The data of all samples are listed in **Supplementary Table 4**.

**Figure 10:**
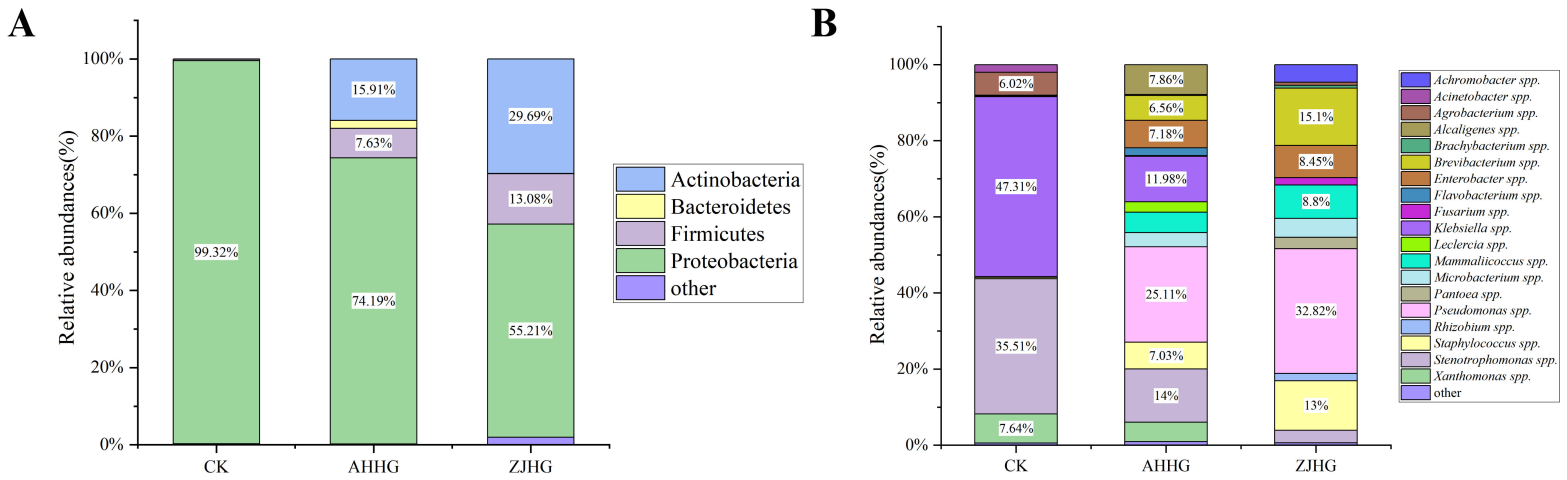
Colony composition of control (CK) and re-inoculated samples at the phylum level **(A)** and genus level **(B)**.

Analysis of the bacterial community composition of CK, AHHG, and ZJHG groups revealed that Proteobacteria was the most dominant bacterial group in all samples. Compared with CK samples, the relative abundance of Proteobacteria decreased in AHHG and ZJHG samples, while the relative abundance of Firmicutes and Actinobacteria increased (**Figure 10A**). The relative changes in abundance of bacterial composition at the genus level between CK, AHHG, and ZJHG showed a decrease in *Klebsiella* spp. and *Stenotrophomonas* spp., and an increase in *Pseudomonas.* spp. (**Figure 10B**).

**Figure 11:**
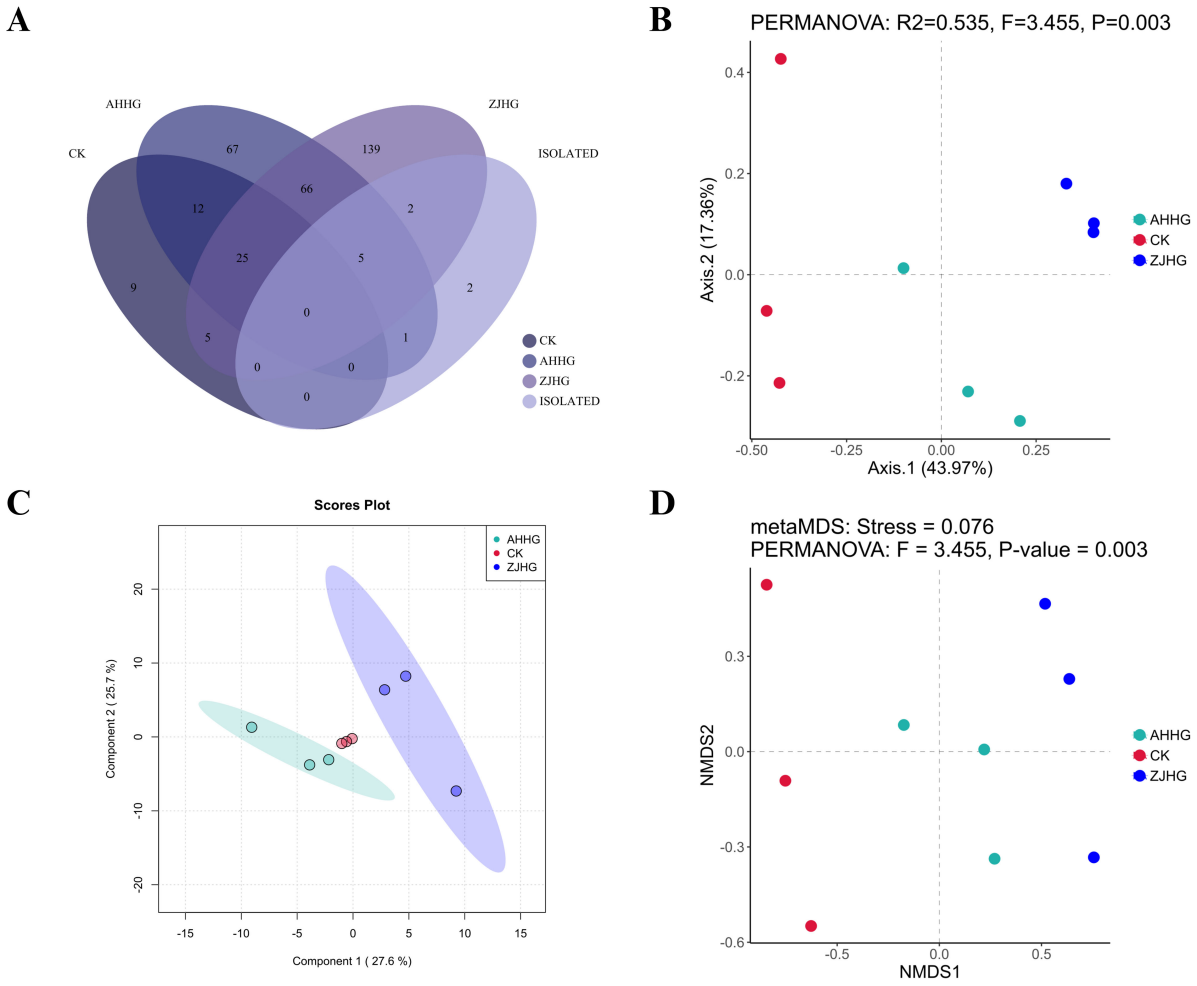
The metagenomic sequencing results of the re-inoculated samples. **(A)** Venn diagram showing the bacterial distribution at the species level between the control (CK) and the cultured metagenomics-separated results (isolated group), as well as the re-inoculated samples AHHG and ZJHG. **(B)** Principal Co-ordinates Analysis (PCoA) of CK and the re-inoculated samples AHHG and ZJHG. **(C)** Partial Least Squares-Discriminant Analysis (PLS-DA) of the CK and re-inoculated samples AHHG and ZJHG. **(D)** Non-metric Multidimensional Scaling (NMDS) analysis of CK and the re-inoculated samples AHHG and ZJHG. Note: the isolated groups refers to the pathogens identified through the pathogenicity test (see sub-section 3.2.5) i.e., *Pseudomonas syringae, P. fulva, P. fluorescens, Pantoea ananatis, Pectobacterium parvum, P. carotovorum, Flavobacterium fluviale, Citrobacter portucalensis, Klebsiella grimontii, Stenotrophomonas maltophilia*.

To screen the changes in bacterial distribution in samples after MBB pathogen re-inoculation, we analyzed the common bacterial species in the CK, AHHG, ZJHG, and isolated groups. As shown in the Venn diagram (**Figure 11A**), a total of 66 bacterial species were found common in AHHG and ZJHG groups, five bacterial species were common in AHHG, ZJHG, and isolated groups. Meanwhile, there were nine bacterial species that only appeared in the CK group. The PCoA analysis was used to compare the similarities or differences between the CK and AHHG and ZJHG groups. The results showed that the bacterial communities between healthy and infected mulberry plants were different, and there were obvious changes at the species-level composition after *P. syringae* strain AHDX, and *P. syringae* strain ZJDX infection (**Figure 11B**). The results of PLS-DA analysis showed a significant difference in the bacterial composition between the CK and AHHG groups. There was a significant difference in the bacterial composition between the CK and ZJHG groups. There was also a significant difference in the bacterial composition between ZJHG and AHHG groups (**Figure 11C**). Non-metric multidimensional scaling (NMDS) analysis showed that the CK, AHHG, and ZJHG groups clustered in a separate cluster (**Figure 11D**).

**Figure 12:**
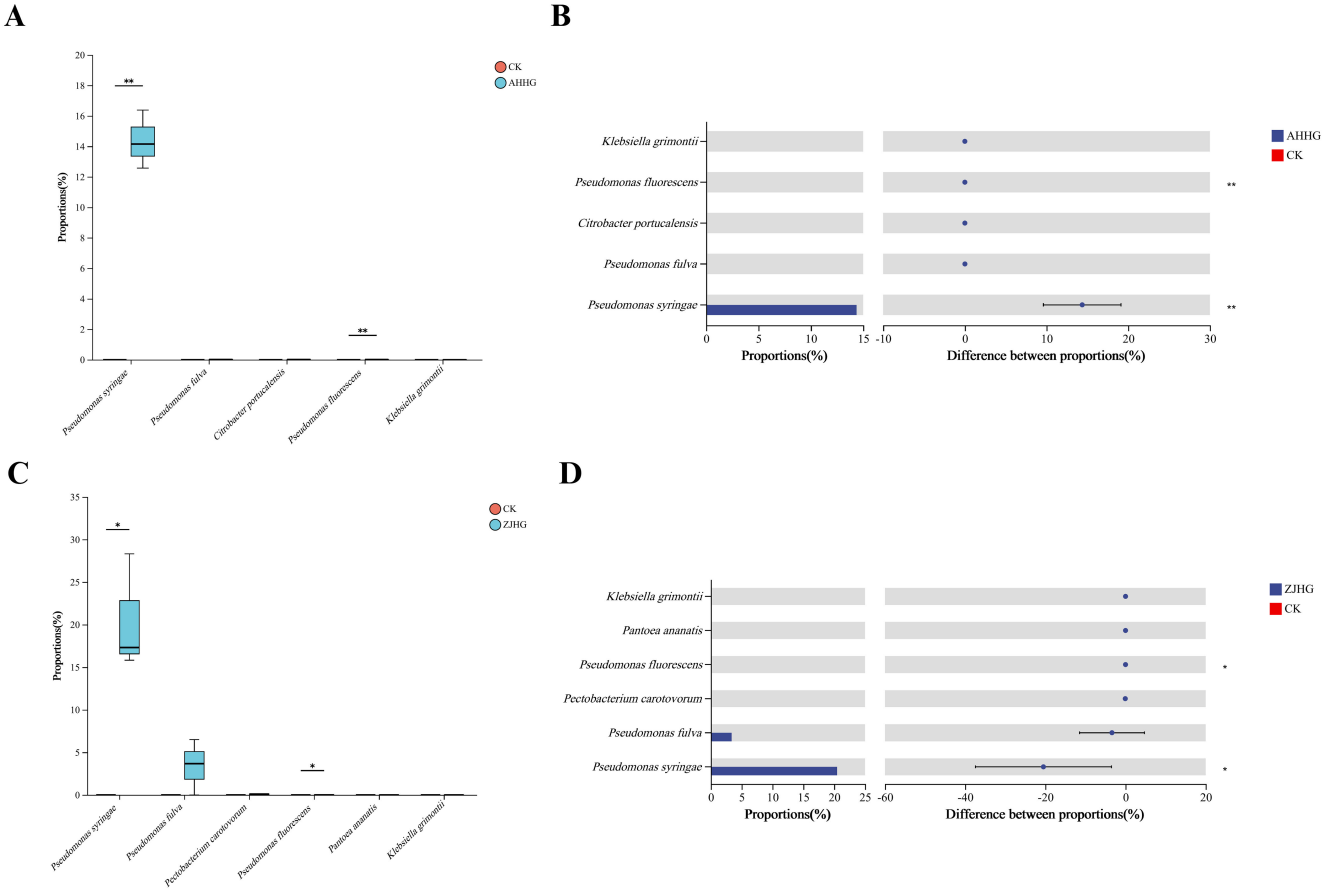
Welch’s t-test of control (CK) against the re-inoculated samples AHHG **(A, B)** and ZJHG **(C, D)**. **(A, C)** The box plots show the relative abundance values of species level that significantly changed relative to the CK in re-inoculated samples on the x-axis, with the y-axis representing the relative abundance values. The box plots display the distribution of expression values or abundance for each feature name across different groups, with different colors representing different groups. **(B, D)** The bar chart on the left shows the average relative abundance of species across different groups on the x-axis, with the y-axis representing different features, and different colors representing different groups. The dot-line plot on the right indicates the difference in average relative abundance of species between the two groups within the specified confidence interval. The value corresponding to the dot represents the difference in average relative abundance, with the dot color indicating the group with the larger abundance. The I-shaped interval above the dot represents the upper and lower limits of the difference. *significant correlation (P<0. 05). **highly significant correlation (P<0. 01).

The Student’s *t* test (*t* test) showed changes in several pathogenic bacteria in the control and re-inoculated groups. The statistical analysis showed that the relative abundance of *P. syringae*, and *P. fluorescens* increased significantly (*P <*0. 01) in the AHHG group compared to the CK group after infection (**Figure 12A**). Based on the color schemes, it was observed that the relative abundance of *P. fulva, C. portucalensis*, and *K. grimonti* increased, but the changes were not significant (*P*>0. 05) (**Figure 12B**). Following ZJHG infection, compared with the CK group, the relative abundance of *P. syringae*, *P. fluorescens*, increased significantly (*P*<0. 05) (**Figure 12C**). The relative abundance of *P. fulva*, *P. carotovorum*, *P. ananatis*, *K. grimonti* also increased but these changes were not significant (*P*>0. 05) (**Figure 12D**).

## 4 Discussion

In the present study, we used combined high-throughput metagenomic sequencing, culturomics, and pathogenicity test (validation by the Koch’s postulates) approaches to analyze the bacterial community composition and diversity in MBB samples collected from different field locations in the eight provinces of China. Based on the omics results and screening of suspected pathogens through pathogenicity test and changes in distribution pattern, *P. syringae*, and *P. fulva* were inferred to be important causal pathogens of MBB in samples analyzed. In addition, microbiome and meteorological data were further analyzed to draw a potential relationship between key causal pathogens and relevant meteorological factors. The identified pathogens showed adaptability to different environments, and that the changes in distribution of causal pathogens of MBB were linked with different climatic conditions of the studied locations.

The MBB samples collected from Jiangsu province showing typical disease samples were used for high-throughput metagenomic sequencing. We found that the microbiome of MBB samples was dominated by bacteria, while eukaryotes (fungi) only accounted for a small proportion (data not shown) of the sequences related to the known taxonomic groups. Given that the classification methods used for identification of microbial community composition based on metagenomic sequencing are based on reference uploaded sequence annotations, and that the reference genomes for most eukaryotes are not available (Bulgarelli et al., 2015), therefore, eukaryote (fungi) sequences weren’t taken into the account. Consistent to finding of our study, a previous study (Xu et al., 2018) using metagenomic sequencing to analyze the microbiome of the citrus rhizosphere, reported that 99. 55% of the non-redundant genes were assigned to prokaryotic (bacteria and archaea), while only 0.17% of the non-redundant genes were annotated as eukaryotic (fungi, protozoa, algae, and plants).

In recent times, high-throughput omics techniques like metagenomic sequencing are increasingly used in the field of plant disease/pathogen research (Tyler et al., 2009). It is said that the “rare” biosphere is actively attracted by specific environments, and may play an important role despite their low abundance (Hartmann and Six, 2023). While the vast majority of microorganisms (bacteria and archaea) referred to as “microbial dark matter” still remains underexplored, the refined high-throughput culturomics techniques have the capacity to illumine the previously unculturable bacterial species (Dickson, 2017). However, use of culturomics is relatively new and substantially lagging behind in plant microbiome research (Sarhan et al., 2019).

It is no surprise that the reduced cost and the level of sequence coverage of samples are the key driving factors for use of these techniques in future studies on complex plant pathogens. Indeed, previously, metagenomic sequencing has been used to study microbial diversity of plant pathogens and to determine causal pathogens of highly destructive plant diseases (Yuan et al., 2023a; Chen et al., 2021). For instance, metagenomic sequencing was used to evaluate the microbial diversity in the phloem of citrus infected with Huanglongbing (HLB) disease (Tyler et al., 2009). Based on annotation results of metagenomic data, *Candidatus* Liberibacter asiaticus was confirmed to be the causal agent of this disease (Tyler et al., 2009). In the present study, the proportion of Proteobacteria in the MBB field samples at the phylum level reached 99. 83%, and *Pseudomonas* spp. also showed a high relative abundance of 99. 24% at the genus level. This finding was consistent with the previous reports from Turkey (Sahin et al., 1999) and Poland (Krawczyk and Łochyńska, 2020), in which *Pseudomonas syringae* was identified as the causal agent of MBB in mulberry plants. We assembled the rRNA sequences of *Pseudomonas* spp. in diseased samples to construct an evolutionary tree, and found that it clustered with *P. syringae* on the same branch. Therefore, based on these results and verification by pathogenicity test, we inferred that *P. syringae* and other *Pseudomonas* spp. are possibly the main causal pathogens of MBB in samples analyzed in the present study.

The bacterial strains obtained through culturomics are suitable for studying bacteria at the strain level (Zhao et al., 2024). In order to improve the sensitivity and accuracy of detection of pathogenic bacteria, we used culturomics technique alongside metagenomic sequencing and classical culture-dependent approaches. This combined approach provided a more comprehensive understanding of the causal pathogens of MBB samples collected from different study locations. Consistent with the metagenomic results, we were able to isolate and identify a large number of bacteria belonging to the phylum Proteobacteria and Firmicutes and Actinobacteria using culturomics technique. While the frequency of isolation of Bacteroidetes was lower, it was consistent with previously reported results (Anteneh et al., 2022; Singh et al., 2017). In slight contrast to the metagenomic sequencing results, the relative abundance of Proteobacteria decreased at the phylum level, while the relative abundance of Firmicutes, Actinobacteria, and Bacteroidetes increased. Previous research on plant-associated microbial community composition has shown that the plant microbial colonization has tissue preference (Edwards et al., 2015). Given that MBB causes lesions in the terminal buds, it is possible that the microbial communities in the samples analyzed in this study maybe similar to the plant phyllosphere microbiome. Tellingly, this microbiome is considered to be more adapted to the environment inside the plant tissues (Leach et al., 2017), and being quite labile to UV radiation, temperature, and sensitivity to drying and dehydration (Bringel and Couée, 2015). We speculate that many other bacteria belonging to Proteobacteria might be present in the samples but they cannot be isolated under the current culture conditions, or they exist in the viable but nonculturable (VBNC) state. It is also possible that the critical factors affecting the cultivation of uncultured plant bacteria and suitable culture/media conditions are not known. Therefore, further research and understanding is required to develop strategies employing multiple and customized media to isolate the diverse bacterial communities.

We compared the results of metagenomic sequencing and culturomics, and identified 10 pathogens (*P. syringae, P. fulva, P. fluorescens, P. ananatis, P. parvum, P. carotovorum, F. fluviale, C. portucalensis, K. grimontii, S. maltophilia*) that were able to infect mulberry and caused typical MBB symptoms, as verified through the Koch’s postulates. Interestingly, most of these bacteria are reported to be common foliar bacteria that cause serious plant diseases, for example, tomato bacterial speck disease (Elsharkawy et al., 2023; Huot et al., 2017), bacterial canker of kiwifruit (Pei et al., 2023), hazelnut decline disease (Lamichhane et al., 2016), and mulberry bacterial blight (Krawczyk and Łochyńska, 2020). Consistent with the previous studies, we reconfirmed that *P. syringae* is indeed the key causal pathogen of MBB (Akhtar and Sarwar, 1988; Krawczyk and Łochyńska, 2020). Similarly, *P. fulva* has been isolated from bananas (Zhang et al., 2022)*, Capsicum annuum L.* bacterial disease (Sheng et al., 2017) and Zanthoxylum black rot (Liu, 2020) disease samples. It has also been reported as an opportunistic human pathogen causing infections such as urinary sepsis (Stark, 2022), bacteremia (Uddin et al., 2018), and wound infections (Cobo et al., 2016). *P. carotovorum* has a global distribution and a wide host range, causing blackleg and tuber soft rot in cruciferous plants and potatoes grown in temperate climates (Czajkowski et al., 2014)*. P. parvum* has been reported to infect potatoes and cause bacterial soft rot, limiting yield and quality, thus affecting global food security (Wang et al., 2022). We have recently reported *P. ananatis* as one of several causal pathogens of mulberry bacterial wilt (Yuan et al., 2023a). However, none of the above bacteria have been reported to cause MBB.

*S. maltophilia* is a common pathogen in nosocomial infections that can cause skin infections in human (Zhong et al., 2023)*. C. portucalensis* (Sellera et al., 2022) and *F. fluviale* (Kämpfer et al., 2012) have been reported as common opportunistic pathogens in air or water and have the potential to become bacteria of public health concern worldwide. Although, these bacteria have not yet been reported as the causal pathogens of mulberry diseases, there is considerable value in studying them, as mulberry and its byproducts are commonly used in human food chain and pharmaceutical industry (Gao et al., 2024; Zhao et al., 2023). Within Enterobacteriaceae, a large group of soft rot Enterobacteriaceae (SRE) bacteria causes blackleg wilt and soft rot diseases in a wide range of important plants worldwide (Pritchard et al., 2016). *Pectobacterium* spp., a sub-group in the SRE, are recognized as the most significant bacterial plant pathogens in terms of economic and yield losses they cause (Pritchard et al., 2016). In the present study, we found that *P. carotovorum* had stronger virulence and caused more serious terminal bud black blight in mulberry than other pathogens under similar conditions. However, *P. carotovorum* was only isolated in one sample. We speculate that, although *P. carotovorum* is one of the important causal pathogens of MBB causing serious blight symptoms, it is possible that its spread to other sampling locations covered in the present study might have been prevented by the fact that it is more adapted to temperate climate zones.

In order to draw a relationship between pathogen distribution, community composition, and relevant metrological factors, we performed the Spearman correlation coefficient analysis to study the distribution of 10 suspected bacterial pathogens of MBB in samples collected from different locations in relation to the local meteorological data of 15 days before and after sampling. The results of the correlation analysis between occurrence of *P. syringae* and meteorological factors suggested that MBB caused by *P. syringae* may occur in summer season with long and rainy days, and that wind and precipitation might help its spread. This is supported by an evidence on bacterial canker of kiwifruit caused by *Pseudomonas syringae* pv. Actinidiae (Donati et al., 2020). It was argued that the bacterial colonization is affected by environmental factors, and that the disease incidence requires a combination of mild temperature and leaf wetness (Donati et al., 2020). Interestingly, in the present study, among all suspected pathogenic bacteria, only *P. syringae* was positively correlated with TEMP, suggesting that it may be more sensitive to environmental temperature. This is also consistent with the report that *P. syringae* has ice nucleation activity (INA) function and can produce ice nucleation proteins to induce ice crystals (Du et al., 2017). Meanwhile, the occurrence of *P. fulva* and *P. fluorescens* was not correlated or negatively correlated with five of the six meteorological factors covered in the present research. This finding suggested that *P. fluorescens* and *P. fulva* might be conditional pathogens endogenous to mulberry or inherent in the air and soil. We argue that these bacteria could cause infection and lead to mulberry blight under specific/favorable changes in conditions i.e., *P. fluorescens* under dry conditions and *P. fulva* under wet conditions. The occurrence of *P. ananatis* was positively correlated with PRES and WDSP, and negatively correlated with RH, PRCP, and TEMP. High-pressure air masses have high density and small gas expansion, which are often accompanied by low temperatures and dryness. We therefore speculate that *P. ananatis* infection is likely to occur in low temperature and dry conditions, environment or season, and wind is also conducive to the spread of *P. ananatis*. The occurrence of *P. parvum, P. carotovorum, F. fluviale, C. portucalensis, K. grimontii* and *S. maltophilia* were all positively correlated with RH and PRCP, suggesting that these pathogens are more adapted to humid and wet environments. Given that the correlation between the pathogen distribution and meteorological factors studied in the present study was based on statistical analysis, therefore, further experimental validation of these relationships may be carried out in the future works. Future research should also focus on exploring the precise mechanistic basis by which these meteorological factors influence the distribution of the causal pathogens of MBB, their virulence and transmission dynamics in different agro-climatic conditions. Studies addressing the evolutionary significance of the identified pathogens in specific areas of adaptation are particularly required to gain more insight in this regard.

Combined metagenomic sequencing and conventional culture methods are presently seen as the “best practice” for the diagnosis of pathogenic microorganisms. Chen et al. (2021) compared the results of metagenomic sequencing and conventional culture methods for diagnosing human infectious pathogens, and reported a highly consistent result between both methods. In the present study, the results of metagenomic sequencing and isolation culture methods were also highly consistent and showed that *P. syringae* and *P. fulva* were widely distributed in the samples. These pathogens also showed high virulence when artificially inoculated in mulberry plants, and therefore, they were considered as the main causal pathogen of MBB. However, further focused studies are still required for confirmation and to gain detailed understanding.

The composition of plant microbial communities is influenced by cooperation, competition, and interactions among countless microbial members. These microbial members as a whole affect plant health (Gao et al., 2021). Interestingly, when we re-inoculated the pathogenic bacteria *P. syringae* to mulberry, the microbial community changed. Infection with MBB pathogens led by *P. syringae* affected the composition of mulberry endophyte community. In support of this, we argue that the increase in the relative abundance of pathogenic bacteria might be proportional to the increase in the endophytic bacteria. However, we currently don’t have concrete evidence to support this notion. We believe that these pathogenic strains may have formed a certain ecological niche in mulberry, posing a threat to overall plant health.

Although, the diversity of experimental methods used in this study helped us report more shared genera between metagenomic sequencing and culturomics, these methods also have limitations. We know that despite obvious advantages of metagenomic technology, its effectiveness is highly dependent on the complexity and biomass of the community, sequencing technology and reference database used. It is therefore possible that certain specific taxa, (e.g. with low abundance) might have been omitted (Li et al., 2023 and references therein). Although culturomics is now seen as an important tool to provide comprehensive insight on the diversity of previously unculturable microbes, it is often considered as a labor/resource-intensive method. Generation of comprehensive strain collections through culturomics is still an unresolved challenge, as it may omit the specific target groups of importance within the microbial community (Li et al., 2023 and references therein).

Tellingly, metagenomics- and Culturomics-based studies have the potential to contribute to agricultural sustainability (Clagnan et al., 2024), we therefore believe that the results presented herein could provide a valuable resource for building climate-smart and resource-efficient mulberry production systems. These results could also serve as a baseline for developing and implementing sustainable approaches like nature-based biological control and integrated pest management to combat pathogens in mulberry production systems. However, if we are to build practically improved mulberry production, future research needs to bridge the experimental data and field application.

## 5 Conclusions

In the present study, we surveyed and collected MBB disease samples in silkworm rearing areas in eight provinces of China, and conducted a comprehensive and in-depth analysis of the potential causal pathogens of MBB. Through combined metagenomic and culturomics techniques, we screened and identified several suspected causal bacterial pathogens of MBB. Based on verification results of pathogenicity test, 10 pathogens including *P. syringae P. fulva, P. fluorescens, P. ananatis, P. parvum, P. carotovorum, F. fluviale, C. portucalensis, K. grimontii and S. maltophilia* were identified as potential causal agents, with *P. syringae* and *P. fulva* being identified as the key pathogens of MBB. Combining the result of isolation of pathogenic bacteria with the meteorological factors, it was found that different bacteria adapt to different environmental conditions, leading to differences in the pathogenic bacteria of MBB in mulberry fields in different climate types and different latitudes. Future focused studies on the causal pathogens of MBB are required to gain insight on the precise infection mechanisms of these pathogenic strains and how they interact/behave in different environments and metrological factors.

## Data Availability

The datasets presented in this study can be found in online repositories. The names of the repository/repositories and accession number(s) can be found in the article/**Supplementary Material**. The raw metagenomic data obtained in this study has been deposited in the National Center for Biotechnology Information (NCBI) GenBank under BioProject number: PRJNA1177990. The genome sequence of pure culture bacteria obtained through isolation and cultivation in this study has been deposited at NCBI GenBank under BioProject number PQ454732-PQ455177.

## Footnotes

1 Statistical data from Ministry of Commerce, People’s Republic of China, https://www.mofcom.gov.cn/).
