# Supplementary figures and images for: Analysis of causal pathogens of mulberry bacterial blight in samples collected from eight provinces of China using culturomics and metagenomic sequencing methods
Source: Front Plant Sci. 2025 Feb 28;16:1517050. doi: 10.3389/fpls.2025.1517050 (PMC11906434; doi:10.3389/fpls.2025.1517050)

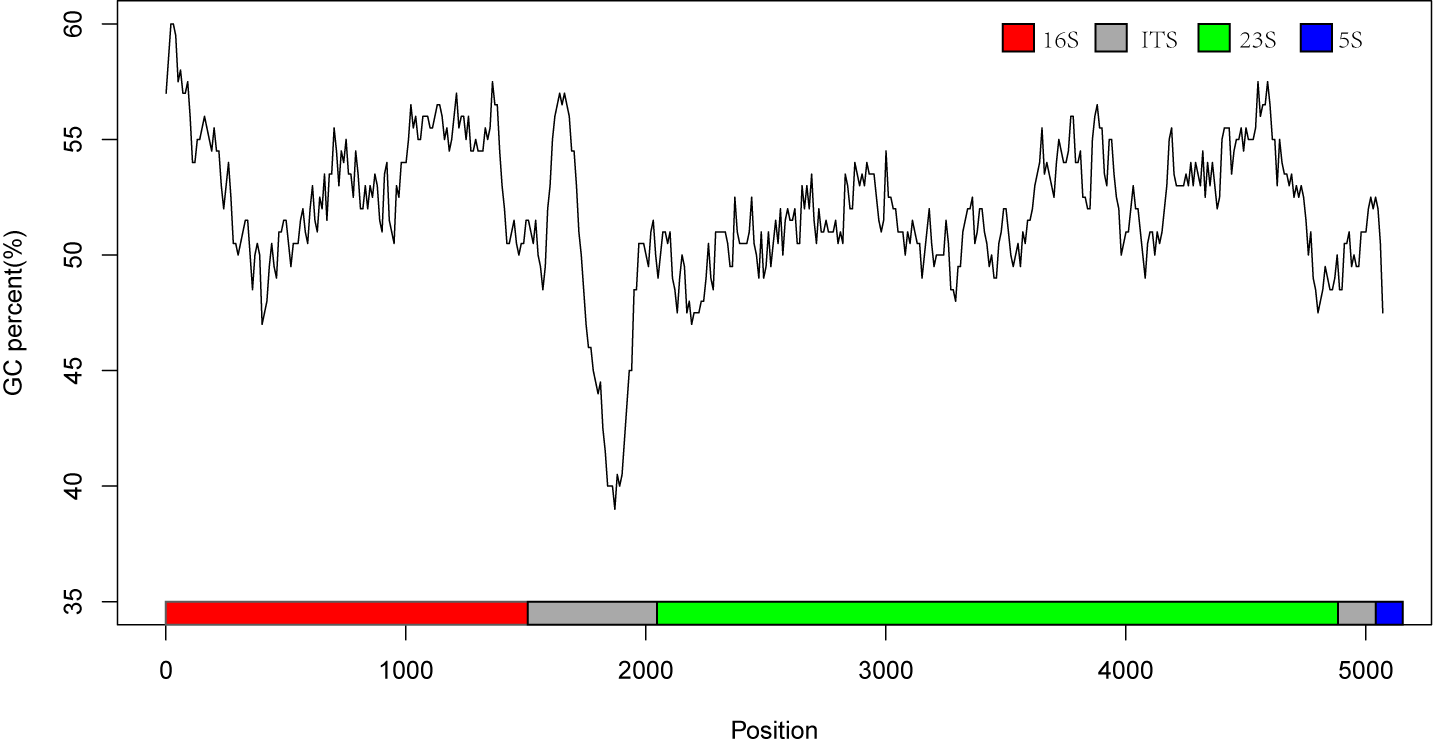


Supplementary Figure 1 rRNA annotation and GC proportion distribution map of *Pseudomonas* spp*.*

Supplement: Supplementary file 1 [file Table1.docx]
