# Supplementary material for: Analysis of causal pathogens of mulberry bacterial blight in samples collected from eight provinces of China using culturomics and metagenomic sequencing methods
Source: Front Plant Sci. 2025 Feb 28;16:1517050. doi: 10.3389/fpls.2025.1517050 (PMC11906434; doi:10.3389/fpls.2025.1517050)
Supplement: Supplementary file 2 [file Table2.docx]

Supplementary table1 Sequencing Data Statistics

|  |  | Length (bp) | Reads | Base (bp) | Total Base (bp) | Q20 | Q30 | GC |
| --- | --- | --- | --- | --- | --- | --- | --- | --- |
| S1 | Read1 | 150 | 15,790,252 | 2,368,537,800 | 4,737,075,600 | 97.16% | 92.34% | 52.55% |
|  | Read2 | 150 | 15,790,252 | 2,368,537,800 |  | 96.36% | 90.78% | 52.87% |

Note: Read1, 2: The first and second reads from paired-end sequencing. Length (bp): Read length of sequencing sequences. Reads: Number of sequencing reads. Base (bp): The number of nucleotide bases sequenced. Total Base Pairs (bp): The total number of base pairs sequenced. Q20: The proportion of base pairs with a quality value greater than 20 (error rate less than 1%) to the total number of base pairs. Q30: The proportion of base pairs with a quality value greater than 30 (error rate less than 0.1%) to the total number of base pairs.

Supplementary table 2 *Pseudomonas* spp*.* rRNA Sequence Information

| **Base** | A | T | G | C | N | Total |
| --- | --- | --- | --- | --- | --- | --- |
| **Number (bp)** | 1,370 | 1,138 | 1,588 | 1,155 | 0 | 5,251 |
| **Percent** | 26.09% | 21.67% | 30.24% | 22.00% | 0.00% | 52.24% |

Supplementary table 3 *Pseudomonas* spp*.* rRNA Assembly Information

| **Region** | **Start** | **End** | **Length(bp)** | **GC (%)** |
| --- | --- | --- | --- | --- |
| 16S | 1 | 1,539 | 1,539 | 54.06 |
| ITS1 | 1,540 | 2,083 | 544 | 47.98 |
| 23S | 2,084 | 4,976 | 2,893 | 52.23 |
| ITS2 | 4,977 | 5,135 | 159 | 50.31 |
| 5S | 5,136 | 5,251 | 116 | 50.86 |
| Total | 1 | 5,251 | 5,251 | 52.24 |

Supplementary table 4 Results of metagenomic sequencing of experimental samples re-infected with *P. syringae.*

| Sample | Raw data |  |  |  |  | Clean data |  |  |  |  |
| --- | --- | --- | --- | --- | --- | --- | --- | --- | --- | --- |
|  | Reads(#) | Base (nt) | GC (%) | Q20 | Q30 | Reads (#) | Base (nt) | GC (%) | Q20 | Q30 |
| AHHG1 | 14781614 | 2217242100 | 47.14 | 97.47;96.83 | 93.27;91.68 | 14305488 | 2145823200 | 47.08 | 97.71;97.19 | 93.58;92.11 |
| AHHG2 | 15810134 | 2371520100 | 52.85 | 97.92;96.03 | 94.13;89.98 | 15350636 | 2302595400 | 52.83 | 98.12;96.58 | 94.41;90.61 |
| AHHG3 | 16826610 | 2523991500 | 54.92 | 98.09;97.07 | 94.67;92.21 | 16244364 | 2436654600 | 54.9 | 98.31;97.47 | 94.96;92.67 |
| CK1 | 17789444 | 2668416600 | 31.83 | 96.86;95.64 | 92.09;89.44 | 17131262 | 2569689300 | 31.75 | 97.17;96.21 | 92.50;90.13 |
| CK2 | 13247304 | 1987095600 | 31.43 | 96.60;95.37 | 91.47;88.85 | 12769796 | 1915469400 | 31.34 | 96.91;95.93 | 91.87;89.53 |
| CK3 | 14456816 | 2168522400 | 36.14 | 97.22;96.14 | 92.84;90.42 | 13970286 | 2095542900 | 36.08 | 97.51;96.69 | 93.23;91.09 |
| ZJHG1 | 21740624 | 3261093600 | 45.93 | 97.71;96.38 | 93.53;90.43 | 21128460 | 3169269000 | 45.82 | 97.87;96.71 | 93.75;90.79 |
| ZJHG2 | 14071700 | 2110755000 | 54.76 | 97.93;96.14 | 94.14;90.16 | 13678356 | 2051753400 | 54.73 | 98.15;96.63 | 94.43;90.72 |
| ZJHG3 | 32796380 | 4919457000 | 49.5 | 98.03;96.76 | 94.47;91.38 | 31651202 | 4747680300 | 49.44 | 98.21;97.08 | 94.72;91.75 |

Note: AHHG1, AHHG2, AHHG3: mulberry samples re-inoculated with *P. syringae* strain AHDX. CK1, CK2, CK: mulberry samples re-inoculated with sterile water. ZJHG1, ZJHG2, ZJHG3: mulberry samples re-inoculated with *P. syringae* strain ZJDX.
